# Early onset multivalvular disease caused by a missense variant in lamin A/C

**DOI:** 10.1016/j.xhgg.2025.100491

**Published:** 2025-08-08

**Authors:** Alexandre Janin, Nathalie Gaudreault, Victoria Saavedra Armero, Zhonglin Li, Ran Xu, Dominique K. Boudreau, Lily Frenette, Julien Ternacle, Danielle Tardif, Sébastien Thériault, Philippe Pibarot, Patrick Mathieu, Christian Steinberg, Yohan Bossé

**Affiliations:** 1 Institut universitaire de cardiologie et de pneumologie de Québec – Université Laval, Quebec City, QC G1V 4G5, Canada; 2 Université Claude Bernard Lyon 1, Université de Lyon, Lyon 69008, France; 3 Department of Molecular Medicine, Université Laval, Quebec City, QC G1V 0A6, Canada

**Keywords:** lamin A/C, multivalvular disease, whole exome sequencing, family study, functional study

## Abstract

Lamins A/C, coded by *LMNA* gene, are crucial for nuclear architecture preservation. Pathogenic *LMNA* variants cause a wide range of inherited diseases called “laminopathies”. A subgroup is referred to “progeroid syndromes” characterized by premature aging and other manifestations including cardiac valve abnormalities. Atypical phenotypes, generally less severe, have also been reported. We report the case of a 26-year-old male with calcific tricuspid aortic and mitral valve diseases. His father was diagnosed with severe aortic valve stenosis and mitral annulus calcification at the age of 38. The goal of this study was to identify the putative variant causing this non-syndromic multivalvular disease. Known disease-causing variants in *NOTCH1*, *FLNA*, and *DCHS1* were first excluded by Sanger sequencing. Whole-exome sequencing was then performed in five family members. A *LMNA* variant (p.Glu262Val) was identified with *in silico* evidences of pathogenicity (CADD [combined annotation dependent depletion] = 33). Cells transfected with the cDNA construct harboring p.Glu262Val were characterized by abnormal nuclear morphology. Along with a literature review, the variant was classified as likely pathogenic. Elucidating the mechanism by which *LMNA* p.Glu262Val specifically affects cardiac heart valves is likely to provide insight about the pathogenesis of Mendelian forms of valvular heart diseases and may help guide the development of therapies.

## Teaser

We report a family with non-syndromic early onset multivalvular disease. Whole-exome sequencing highlighted an LMNA missense variant (p.Glu262Val). *In silico* algorithms and functional studies demonstrate the variant pathogenicity. This article underlines the well-known phenotypic heterogeneity of laminopathies and supports the hypothesis of a disease linked to *LMNA* variants.

## Introduction

Population-based genomic approaches are starting to elucidate the genetic component of valvular heart diseases (VHD).^1^^,^^2^^,^^3^ There are however only a few non-syndromic genes known to cause Mendelian forms of VHD including notch receptor 1 (*NOTCH1*) associated with developmental valve anomalies and severe valve calcification,^4^ filamin A (*FLNA*) associated with myxomatous valvular dystrophy,^5^ and dachsous cadherin-related 1 (*DCHS1*) associated with mitral valve prolapse.^6^ These genes were identified by the presence of more than one variant known to cause the disease and further investigations in other families and sporadic cases have revealed additional likely pathogenic variants.^7^^,^^8^ Screening for variants in these three genes is thus a prerequisite in genetically suspected cases of early onset valve disease.

In this study, we evaluated a single family characterized by premature multivalvular disease. A Mendelian autosomal-dominant inheritance was suspected, but the affected cases were free of known disease-causing variants in *NOTCH1*, *FLNA*, and *DCHS1*. The goal of this study was to identify the putative causal variant and gene.

## Material and methods

### Ethics statement

All individuals provided written informed consent, and the ethics committee of the Institut universitaire de cardiologie et de pneumologie de Québec – Université Laval approved the study (no. 20341).

### DNA and clinical assessment

Three family members visited our research institution for an echocardiogram and genetic testing. The paternal aunt sent a blood sample and echocardiogram report from another institution. Blood from the deceased father was not available; however, a formalin-fixed paraffin-embedded skin melanoma biopsy was shipped to our laboratory. Sample processing and DNA extraction are provided in the supplemental information.

### WES

Libraries were generated using the SureSelectXT Low Input Automated Target Enrichment for Illumina Paired-End Multiplexed Sequencing (Agilent) and using SureSelect Human All Exon V7 as per the manufacturer’s recommendations. Captured libraries were quantified using the Kapa Illumina GA with Revised Primers-SYBR Fast Universal kit (Kapa Biosystems). Average size fragment was determined using a LabChip GX (PerkinElmer) instrument. DNA sequencing was performed on the Illumina NovaSeq 6000 S4 flow cell.

### *In silico* filtering of the putative variant

Allele frequencies of identified genetic variants were compared with publicly available databases including the Genome Aggregation Database (gnomAD version 4),^9^ and the variant browser Bravo from the NHLBI’s TOPMed program (https://bravo.sph.umich.edu/). Pathogenicity of missense variants was evaluated using PolyPhen,^10^ AlphaMissense,^11^ and the CADD framework.^12^ To evaluate the effect on splicing, two algorithms were used: MaxEntScan^13^ and SpliceAI.^14^ The ACMG (American College of Medical Genetics and Genomics) guidelines were used for the interpretation of variants.^15^

### Vectors construction, transfection, and nuclear morphology assessment

The expression vector containing the wild-type (WT) *LMNA* cDNA (NM_170707) sequence fused to green fluorescent protein (GFP) was purchased from SinoBiological (Ref HG12058-ACG, Vector backbone pCMV3-C-GFPSpark). E262V and D300G plasmids were generated by site-directed mutagenesis using the Q5 Site-Directed Mutagenesis Kit (New England Biolabs). Sanger sequencing of plasmids and primers are in supplemental information.

HEK293T cells were seeded at 1.3 × 10^5^ cells/well in a 6-well plate with 2 mL growth medium the day before transfection. Each plasmid (2 μg) was transfected using Lipofectamine 3000 (Invitrogen) as per the manufacturer’s instructions. After 72 h, cells were passed into medium containing 25 μg/mL hygromycin B (Sigma-Aldrich) for selection. Approximately 2 weeks later, single colonies were isolated and then expanded to make stable cell lines.

Cells were fixed with 4% paraformaldehyde for 10 min, washed with PBS, permeabilized in PBS with 0.1% Triton X-100 for 30 min, and blocked in PBS containing 1% BSA and 10% FBS for 30 min. Nuclei were stained with DAPI for 30 min. Immunofluorescent images were acquired with a Zeiss LSM800 Axio Observer Z1 microscope (ZEN software, version 2.3) and analyzed with ImageJ (version 1.54). Nuclear shape parameters (area, perimeter, and nuclear contour ratio) were estimated by ImageJ “Analyze Particles” plugin with default parameters (version 1.54). Juxtaposed or overlapping nuclei were manually corrected. Automatic thresholding of the 8-bit images was performed. Parameters included a minimum size of 10 pixels, circularity of 0.1–1.0, and exclusion of edge-touching particles. The nuclear contour ratio, defined as 4πA/P^2^, where A is the nuclear area and P is the perimeter, was calculated to assess circularity (1 = perfect circle). Results are expressed as mean ± standard deviation.

## Results

### Family history

**Figure 1:**
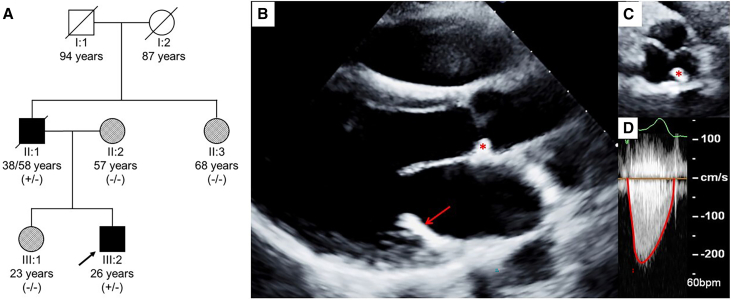
Family pedigree and phenotype (A) Family pedigree. Each symbol indicates a female (circle) or male (square) family member. Black symbols indicate affected individuals, gray ones indicate unaffected family members and white ones indicate unphenotyped relatives. The text immediately below the symbols gives the family member ID; age at the echocardiogram evaluation or age of death when applicable (presented as age of diagnosis/age of death); and the *LMNA* Glu262Val variant status. A diagonal line across symbol indicates that the individual is deceased. The index case (black arrow) of this family is individual III:2. (B–D) Two-dimensional echocardiography of the proband. (B) Parasternal long-axis view. (C) Parasternal short-axis view of the aortic valve. (D) Transaortic valvular flow velocity measured by continuous-wave Doppler ultrasound. Red star: calcification of the base of the left-coronary aortic valve cusp. Red arrow: thickening and calcification of the posterior mitral valve leaflet.

A family was referred to us with non-syndromic early onset multivalvular disease. The mother reported that her husband had severe aortic valve and mitral annulus calcification diagnosed at the age of 38. He had succumbed from the disease at 58 after several cardiac surgeries. The severe multivalvular phenotype at this young age remained unexplained. After her husband passed away, her 26-year-old son (proband) and a 23-year-old daughter, underwent an echocardiography screening. He had normal appearance with no sign of premature aging, skin disease, or dysmorphic feature. However, the transthoracic echocardiography revealed isolated mild aortic valve stenosis (aortic valve area of 1.39 cm^2^, transvalvular peak velocity of 2.1 m/s, and transvalvular peak and mean gradients of 18 and 11 mmHg, respectively) and mild mitral valve regurgitation likely related to leaflet thickening. The thickness of the anterior mitral leaflet was 4.7 mm (Figure 1). The left ventricular ejection fraction (56%) and left ventricular end diastolic diameter (50.1 mm) were preserved. The echocardiography findings mirrored the phenotype documented in his father’s medical record. The proband’s mother, sister, and paternal aunt had normal echocardiogram. None of the family members displayed features of a dilated cardiomyopathy or atrioventricular conduction defects. The clinical presentation suggested an autosomal-dominant inheritance. A genetic investigation was thus initiated to identify the molecular cause of valve disease. Accordingly, the son in this report is considered the proband (Figure 1). A two-step strategy was used to identify the genetic etiology, first testing candidate genes and then whole-exome sequencing (WES).

### Genetic testing

Genetic testing excluded known variants causing valve diseases in *NOTCH1*, *FLNA*, and *DCHS1* (see supplemental information and Figure S1). WES was performed in the five family members. Steps to call and filter genetic variants are depicted in Figure S2. Briefly, by combining the VCF files of the five individuals, 61,518 exonic variants were identified. After the exclusion of synonymous variants, we kept variants shared between the proband and the father and absent from the mother, sister, and paternal aunt. The 241 remaining variants were further filtered based on minor allele frequencies of <0.1% in publicly available databases and a pathogenicity CADD score of at least 10. The 15 remaining variants are indicated in Table 1. Among them, 2 are not represented in gnomAD version 4. The variant located in *MRM3* is predicted to be benign based on PolyPhen and AlphaMissense, whereas the variant in *LMNA* is predicted to be damaging by pathogenicity scores (Table 1). Taking together, the evidence suggests that the candidate pathogenic variant is in the *LMNA* gene, which we prioritized in downstream follow-up experiments.

**Table 1:** List of candidate variants from the whole-exome sequencing

Variation	Consequence	Gene [OMIM]	Existing variation	gnomAD NFE AlleleFreq	gnomAD allele count and AlleleFreq	TOPMed (allele count)	CADD PHRED v.1.7	PolyPhen HVAR Score and prediction	AlphaMissense Score and class
1_52810458_G/A	missense	*ZFYVE9* [603755]	rs140709371	0.0002405	234, 0.0008291	148 2 HMZ	28.5	0.704 possibly damaging	0.085 likely benign
1_153916583_C/T	missense	*DENND4B* [619843]	rs200116588	0.00002432	48, 0.0001774	119 no HMZ	21.3	0.008 benign	0.082 likely benign
**1_156104741_A/T**	**missense**	***LMNA*** [150330]	**–**	**–**	–	**0**	33	**0.987** **probably damaging**	**0.945** **likely pathogenic**
1_161987291_T/C	missense	*OLFML2B*	rs145092080	0.0002971	193, 0.0006945	174 no HMZ	20.4	0.294 benign	0.076 likely benign
2_60773406_C/G	missense	*BCL11A* [606557]	rs150125298	0.0003891	160, 0.0005671	134 no HMZ	24.3	0.247 benign	0.191 likely benign
5_68616225_G/A	missense	*CCDC125* [613781]	rs111417600	0.00003516	76, 0.0003022	42 no HMZ	24.0	0.962 probably damaging	0.123 likely benign
7_38803086_A/G	missense	*VPS41* [605485]	rs148532572	0.001148	202, 0.0007250	189 no HMZ	23.6	0.033 benign	0.698 likely pathogenic
10_72511880_C/T	missense	*ADAMTS14* [607506]	rs142585866	0.0002828	234, 0.0008354	290 no HMZ	34	0.932 probably damaging	0.52 ambiguous
11_5529215_C/T	missense	*UBQLN3* [605473]	rs147771363	0.0004595	308, 0.001093	433 3 HMZ	21.6	0.02 benign	0.067 likely benign
14_19378000_A/G	missense	*OR11H12*	rs761048370	0.0007870	187, 0.0008659	2366 0 HMZ	22.4	0.995 probably damaging	0.176 likely benign
16_22358755_G/A	missense	*CDR2* [117340]	rs372728039	0.00001556	14, 0.00004968	12 no HMZ	16.51	0.007 benign	0.094 likely benign
16_22545898_A/C	missense	*NPIPB5*	rs774565848	0.000007964	19, 0.00007549	439 1 HMZ	8.709	0.923 probably damaging	0.45 ambiguous
17_686469_A/C	missense	*MRM3,* *RNMTL1*	–	–	–	0	23.4	0.262 benign	0.144 likely benign
18_29788170_G/A	stop gained	*MEP1B* [600389]	rs200474192	0.0001402	179, 0.0006378	151 1 HMZ	42	–	–
20_62221499_C/A	missense	*GMEB2* [607451]	rs756634351	0.00002987	13, 0.00005956	14 no HMZ	19.21	0.006 benign	0.084 likely benign

The *LMNA* variant identified in this study is in bold.

### *In silico* evaluation of the *LMNA* variation

**Figure 2:**
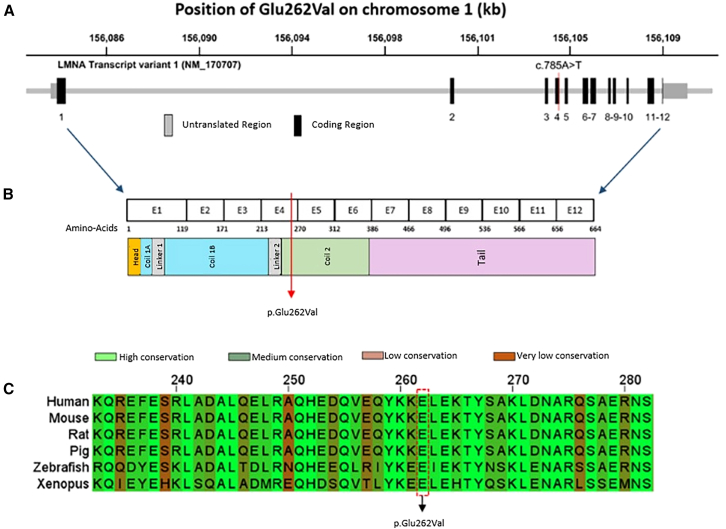
Variant p.Glu262Val in *LMNA* (A) Exon-intron structure of *LMNA*. Red vertical line indicates the variant p.Glu262Val, c.785A>T. (B) Corresponding protein domains. Red arrow indicates the p.Glu262Val variant. (C) Alignment of LMNA amino acid sequences across species. LMNA, Lamin A/C.

The proband and the father are carriers of a missense variant in *LMNA* (Figure 2A). This variant in exon 4 (NM_170707.4:c.785A>T, p.Glu262Val) replaces glutamic acid (acidic) with valine (nonpolar) at codon 262. It has not been reported in the ClinVar database and is predicted to be damaging by PolyPhen2 (0.987), CADD (33.0), AlphaMissense (0.945), and Rare Exome Variant Ensemble Learner (0.877) algorithms (Tables 1 and S1). The Glu262 residue, located in the coil 2 domain (Figure 2B), is highly conserved across species (Figure 2C). A variation affecting the same amino acid (p.Glu262Lys) has been reported as “likely pathogenic” in the ClinVar database and associated with a *LMNA*-related phenotype (Hutchinson-Gilford progeria syndrome, ID: 1698456). Sanger sequencing (Figure S3) confirmed the exome sequencing result.

Additionally, *in silico* analysis showed that the substitution created a relatively strong donor splice site in exon 4. According to MaxEntScan, the *de novo* donor splice site shows a close strength (4.0) to the natural donor splice site (4.7). SpliceAI algorithms confirmed the potential creation of *de novo* donor splice site (donor gain = 0.91).

Based on these *in silico* data, and given a low rate of benign missense variations in *LMNA*, the variant should be considered likely pathogenic according to the ACMG classification (PP2, PM2, PM5, and PP3).^15^ *In vitro* studies were undertaken to assess effects on splicing and nuclear architecture to better validate pathogenicity.

### Functional assays

**Figure 3:**
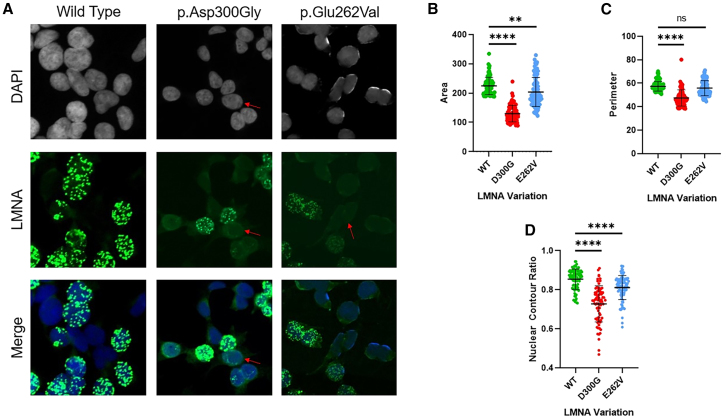
Nuclear abnormalities in p.Glu262Val transfected cells (A) Fluorescence of nuclei shape from a negative control (wild-type), a positive control (p.D300G) and our variant of interest (p.E262V). Arrows indicate abnormal lamin A/C localization. (B–D) ImageJ analysis of nuclear morphology for area (B), perimeter (C), and nuclear contour ratio (D). Unpaired t test was performed using GraphPad Prism version 8.0.1 (ns, not significant; ∗*p* < 0.05, ∗∗*p* < 0.01, ∗∗∗*p* < 0.001, ∗∗∗∗*p* < 0.0001). The results are shown as mean ± standard deviation.

Minigene reporting splicing assays demonstrated that p.Glu262Val was not associated with any splicing effects in our experimental workflow (see supplemental information and Figure S4). In contrast, the fluorescence analysis of nuclei from HEK293T transfected with LMNA-GFP plasmids showed abnormal morphology, as previously described for the p.Asp300Gly variant, used as positive control^16^^,^^17^ (Figure 3A). Images were analyzed to determine three nuclear parameters: area, perimeter, and nuclear contour ratio. We evaluated 90 nuclei for the WT condition, 76 for the p.Asp300Gly variant, and 94 for the p.Glu262Val variant.

Relative to the WT transfected cells, p.Asp300Gly and p.Glu262Val nuclei had significantly smaller nuclear areas than controls (Figure 3B, 129.9 ± 28.1, *p* < 0.0001 for p.Asp300Gly and 203.9 ± 49.7, *p* = 0.009 for p.Glu262Val versus 224.4 ± 29.7 for WT). Although nuclei perimeters were not significantly different for p.Glu262Val (55.9 ± 6.5, *p* = 0.053) compared with WT (57.4 ± 4.0), it was the case for p.Asp300Gly (47.5 ± 6.9, *p* < 0.0001) (Figure 3C). Finally, significant differences were found in the nuclear contour ratio between p.Asp300Gly and WT (0.73 ± 0.09 versus 0.85 ± 0.05, *p* < 0.0001) and between p.Glu262Val and WT (0.81 ± 0.06 versus 0.85 ± 0.05, *p* < 0.0001) (Figure 3D). The area and perimeter for the positive control variant were as reported previously.^18^ Based on the results of *in vitro* assays, the PS3 argument of the ACMG classification could be added.^15^

### Previously reported *LMNA* variants in valvular diseases

To date, 15 *LMNA* variants have been associated with a valvular phenotype in the literature and in the HGMD database (Tables S1 and S2). The variants reported in classical Hutchinson-Gilford progeria syndrome (HGPS) and Werner syndrome (WS) were excluded, given the more severe phenotype of these diseases. All of these are missense variants in a heterozygous state. All are also absent in gnomAD database (version 4) and the majority (13/15) are considered as likely pathogenic or “pathogenic” according to ACMG guidelines. These variants are spread all over the gene. Only six were found in patients with valvular calcifications (Table S2). Two variants (p.Glu138Gln and p.Glu145Gly) are associated with non-syndromic valvular features and no other abnormalities such as premature aging, skin changes, neuromuscular, or metabolic phenotypes.^17^ These are located in the coil 1A region, whereas the reported variant is located at the beginning of the coil 2 domain (Table S1).

## Discussion

The indication for genetic testing in this study was a familial form of early onset aortic valve and mitral annulus calcification that was likely transmitted in an autosomal-dominant mode. After ruling out variants known to cause non-syndromic valve disease in *NOTCH1*, *FLNA*, and *DCHS1*, WES was performed. We identified a missense variant, p.Glu262Val, in *LMNA* with prediction of damaging effect by *in silico* testing.

*LMNA* encodes the A-type nuclear lamins (lamin A and lamin C), intermediate filament proteins essential for the nuclear envelope’s structural integrity. Hundreds of *LMNA* variants cause laminopathies, which manifest in diverse phenotypes, including premature aging and different forms of cardiomyopathy, muscular dystrophy, lipodystrophy, and neurodegenerative disorders.^19^ Interestingly, cardiac valve calcification and dysfunction are clinical features observed in HGPS, WS, and atypical progeroid syndromes, which exhibit milder phenotypes compared with classic forms.^20^ Furthermore, two variants (p.Glu138Gln and p.Glu145Gly) have been associated with non-syndromic valvular abnormalities.^17^ This study emphasizes the well-known heterogeneity of laminopathies.^19^ Moreover, deciphering the molecular mechanisms underpinning the distinct and shared clinical manifestations of the variations is likely to provide more insights about the pathogenesis of valve disease and may help us to guide the development of valve-targeting therapies.

Tissue-specific manifestations of *LMNA* variants is a well-known phenomenon in cardiomyopathies.^21^^,^^22^ A *LMNA* heterozygous splice-site variant causing cardiac disease showed no molecular or nuclear abnormalities in patient fibroblasts.^23^ Reduced *IGFBP5* mRNA levels in these fibroblasts may mask the phenotype in unaffected tissues, while this compensatory mechanism might not occur in cardiac tissues, making them more vulnerable to LMNA haploinsufficiency. Another study evaluated the pro-osteogenic effects of *LMNA* variants in four types of primary human cells of mesenchymal/cardiovascular origin, including human aortic valve interstitial cells.^22^ Variant-dependent effects on the expression of osteogenic markers in response to lipopolysaccharide or an osteogenic differentiation medium were observed, but more interestingly the effect of each *LMNA* variant was strongly dependent on the cell type. This raises the possibility that the *LMNA* p.Glu262Val variant may act specifically in heart valve cells, potentially explaining the observed clinical phenotype in affected relatives.

Taken together, the ACMG criteria^15^ that support the *LMNA* p.Glu262Val as a likely pathogenic variant and the cause of early onset multivalvular disease in this family are: the candidate missense variant is absent from controls (PM2), novel missense change at an amino acid residue where a different missense change determined to be pathogenic has been seen before (PM5), multiple lines of computational evidence support a deleterious effect on the gene product (PP3), and a low rate of benign missense variant in *LMNA* (PP2). If the nuclear morphology defects observed in our experiments are confirmed by other studies, *in vitro* functional studies supportive of a damaging effect on the protein (PS3) could be added, and the variant classified as pathogenic.

We cannot totally exclude the existence of other disease-causing or disease-modifying variants in this family. We have identified additional candidate variants shared between the proband and the affected father (Table 1). We have also identified a missense *DCHS1* variant (p.S415R). Different variants in this gene have been shown to cause mitral valve prolapse^6^ and we previously observed an enrichment of S415R in sporadic cases of mitral valve prolapse.^8^ However, its minor allele frequency (1% in individuals of European ancestry) is relatively high making a pure Mendelian disease-causing effect unlikely. Unfortunately, no additional family members are available to delineate the cosegregation of disease with *LMNA* p.Glu262Val, *DCHS1* p.Ser415Arg, and other candidate variants. Thus, a two-hit or multi-hit etiology cannot be excluded in this family.

Some limitations should be mentioned. We reported only one family with few living relatives, restricting the segregation analysis to two affected individuals. The follow-up of the relatives is limited and the curation of variants identified by WES is based on the phenotype observed at the time of the study. We assessed the variant’s functional impact via nuclear structure analysis. Since lamin A and GFP-tagged protein overexpression are known to induce aggregates, we analyzed nuclear morphology independently of them. Nonetheless, we cannot fully exclude the possibility that aggregates may have influenced our findings. For this reason, the PS3 argument was not added to our final interpretation.

In conclusion, we identified a *LMNA* missense variant, p.Glu262Val associated with non-syndromic multivalvular disease. It provides insight into the valve diseases pathogenesis and the specificity of p.Glu262Val to cause exclusively cardiac valve manifestations is of interest. Further work is needed to determine the role of *LMNA* in the development of valve diseases and elucidate the targeted impact of p.Glu262Val in valve tissues.

## Data and code availability

The datasets supporting the current study have not been deposited in a public repository in accordance with the informed consent and institutional ethics approval, but are available from the corresponding author on request.

## Acknowledgments

We are grateful to all members of this family for their proactive collaboration. P.P. holds the Canada Research Chair in Valvular Heart Disease and his research program is supported by a Foundation Scheme Grant from 10.13039/501100000024Canadian Institutes of Health Research (Ottawa, Ontario, Canada). P.M. is the recipient of the Joseph C. Edwards Foundation granted to Université Laval. C.S. holds a Junior 1 Clinical Research Scholar award from the 10.13039/501100000156Fonds de Recherche du Québec - Santé (FRQS). Y.B. holds a Canada Research Chair in Genomics of Heart and Lung Diseases. This work was supported by the Heart and Stroke Foundation of Canada, the 10.13039/501100000024Canadian Institutes of Health Research (PJT–153396, PJT–159641), and the 10.13039/501100000156FRQS.

## Declaration of interests

The authors declare no competing interests.

## Supplemental information

Document S1. Figures S1–S5 and Tables S1–S5

Document S2. Article plus supplemental information
