# Supplementary material for: Early onset multivalvular disease caused by a missense variant in lamin A/C
Source: HGG Adv. 2025 Aug 8;6(4):100491. doi: 10.1016/j.xhgg.2025.100491 (PMC12398237; doi:10.1016/j.xhgg.2025.100491)
Supplement: Document S1. Figures S1–S5 and Tables S1–S5 [file mmc1.pdf]

HGGA, Volume *6*

Supplemental information

Early onset multivalvular disease caused by a missense variant in lamin A/C

Alexandre Janin, Nathalie Gaudreault, Victoria Saavedra Armero, Zhonglin Li, Ran Xu, Dominique K. Boudreau, Lily Frenette, Julien Ternacle, Danielle Tardif, Sébastien Thériault, Philippe Pibarot, Patrick Mathieu, Christian Steinberg, and Yohan Bossé

Supplemental results

Candidate genes testing

Genetic testing was successful to exclude known mutations causing valve diseases in *NOTCH1* *FLNA* and *DCHS1* (**Figure S1**). However, the proband and his father carried a *DCHS1* missense variant in exon 2 (rs117368891, c.1245C>A, p.Ser415Arg), resulting in a substitution of serine with arginine at position 415. Inherited from the father, this S415R variant was absent in unaffected family members, including the mother, paternal aunt, and sister. The proband's heterozygous genotype aligns with dominant inheritance, though segregation data is limited. The strongest evidence of pathogenicity for this variant comes from our previous study[6]. We have recently sequenced the coding regions of *DCHS1* in 100 patients with mitral valve prolapse and three of them were carriers of S415R (3%). This represents an enrichment of 5 to 10-fold compared to reference populations (0.3 to 0.7%). However, the allele frequency is still relatively high (1.08% in non-Finnish Europeans of gnomAD v2.1.1). Some computational algorithms like PolyPhen (1) and CADD (18.93) suggest a deleterious effect, while AlphaMissense classifies it as “ambiguous” (pathogenicity score: 0.408). ClinVar reports the variant six times (ID: 445814), mostly as “benign” or “likely benign”. Overall, the evidence is insufficient to confirm S415R as a disease-causing variant for the patient’s phenotype.

Minigene reporter splicing assay

To evaluate the functional impact on splicing, wild-type (WT) and mutated minigene constructs containing exon 4 of *LMNA* gene and its intronic boundaries were built. After total RNA extraction, RT-PCR amplification of WT and mutated construct resulted in 418 bp products corresponding to normal transcripts, which were further confirmed by Sanger sequencing

(**Figure S4**). Given that the lengths of the transcripts were the same between WT and mutated conditions, the minigene assay confirmed that this exonic variation was not associated with any splicing defects.

A) *NOTCH1*

NOTCH1 R1107X rs41309764	**Proband (son)**	**Homozygote C/C**	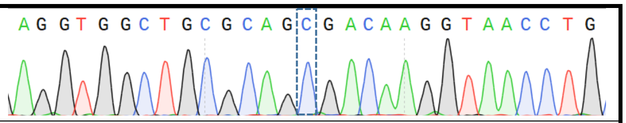
	**Father**	**Homozygote C/C**	Determined by Exome Sequencing
	**Mother**	**Homozygote C/C**	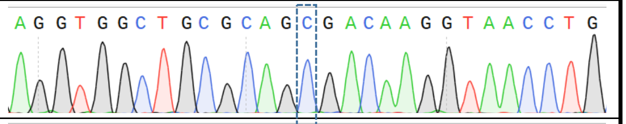
	**Sister**	**Homozygote C/C**	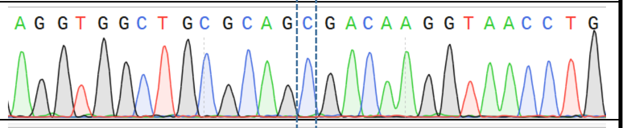
	**Paternal aunt**	**Homozygote C/C**	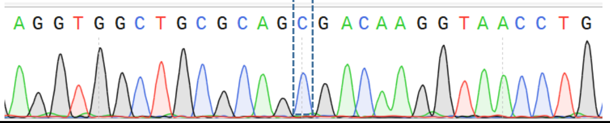

NOTCH1 H1504del rs41309766	**Proband (son)**	**Homozygote G/G**	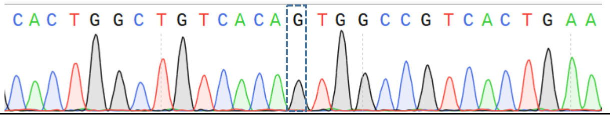
	**Father**	**Homozygote G/G**	Determined by Exome Sequencing
	**Mother**	**Homozygote G/G**	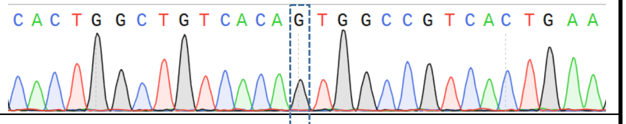
	**Sister**	**Homozygote G/G**	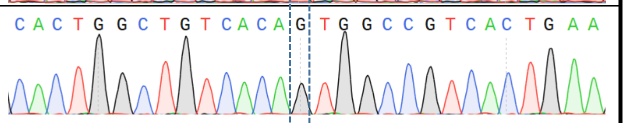
	**Paternal aunt**	**Homozygote G/G**	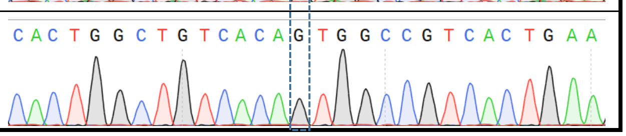

B) *FLNA*

***FLNA* G288R rs267606816**	**Proband (son)**	**Homozygote G/G**	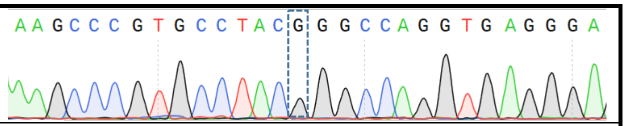
	**Father**	**Homozygote G/G**	Determined by Exome Sequencing
	**Mother**	**Homozygote G/G**	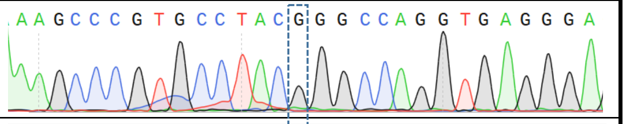
	**Sister**	**Homozygote G/G**	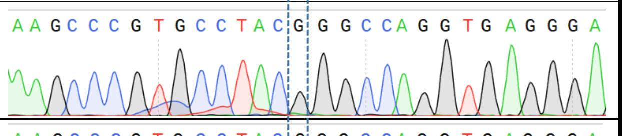
	**Paternal aunt**	**Homozygote G/G**	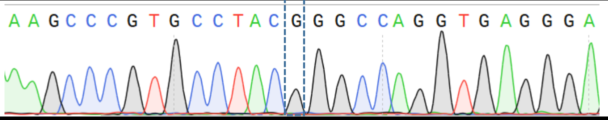

***FLNA* P637Q rs267606815**	**Proband (son)**	**Homozygote G/G**	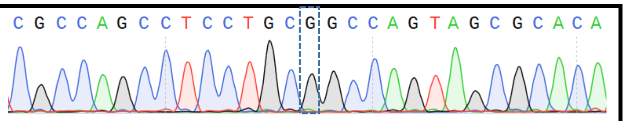
	**Father**	**Homozygote G/G**	Determined by Exome Sequencing
	**Mother**	**Homozygote G/G**	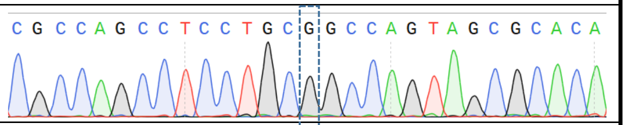
	**Sister**	**Homozygote G/G**	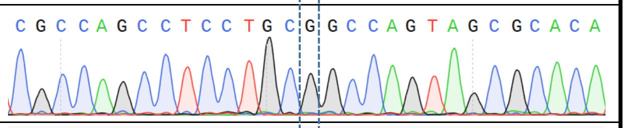
	**Paternal aunt**	**Homozygote G/G**	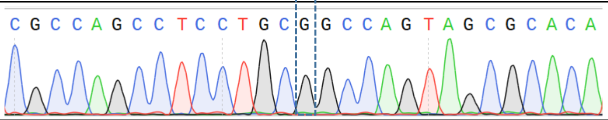

FLNA V711D rs267606817			
	**Proband (son)**	**Homozygote A/A**	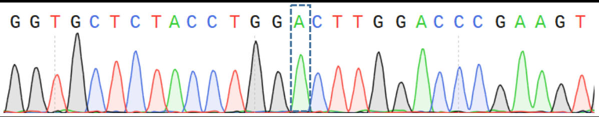
	**Father**	**Homozygote A/A**	Determined by Exome Sequencing
	**Mother**	**Homozygote A/A**	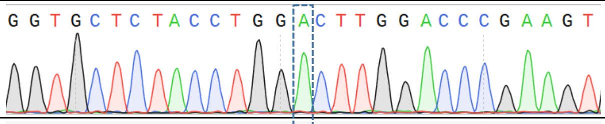
	**Sister**	**Homozygote A/A**	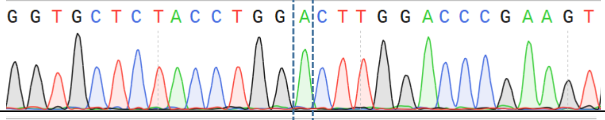
	**Paternal aunt**	**Homozygote A/A**	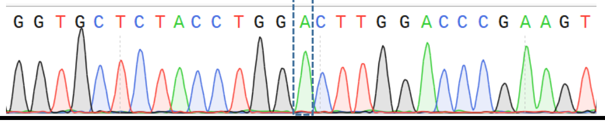

FLNA 1944 bp deletion no rs #			
	**Father**	**Homozygote 2933 bp**	Determined by Exome Sequencing
	**Proband (son)**	**Homozygote 2933 bp**	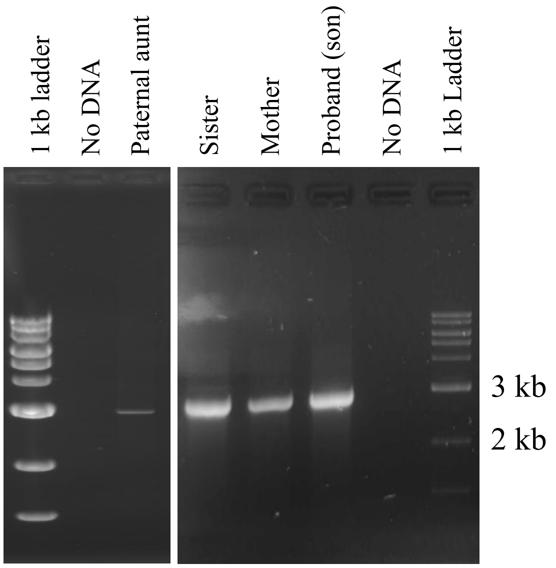
	**Mother**	**Homozygote 2933 bp**	
	**Sister**	**Homozygote 2933 bp**	
	**Paternal aunt**	**Homozygote 2933 bp**	

C) *DCHS1*

***DCHS1* S415R rs117368891**	**Proband (son)**	**Heterozygote G/T**	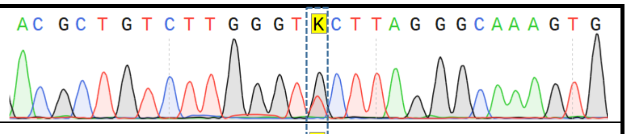
	**Father**	**Heterozygote G/T**	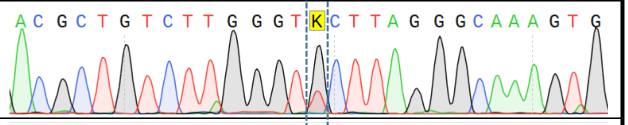
	**Mother**	**Homozygote G/G**	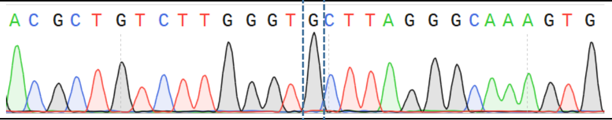
	**Sister**	**Homozygote G/G**	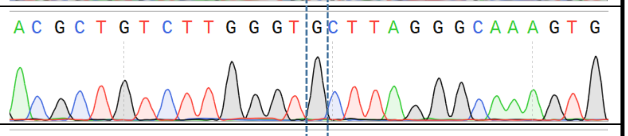
	**Paternal aunt**	**Homozygote G/G**	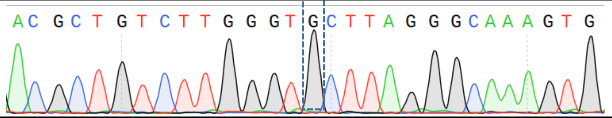

***DCHS1* R2330C rs768737101**	**Proband (son)**	**Homozygote G/G**	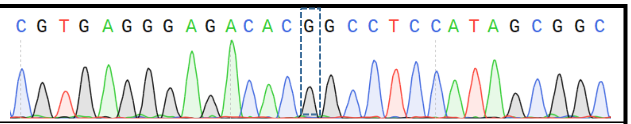
	**Father**	**Homozygote G/G**	Determined by Exome Sequencing
	**Mother**	**Homozygote G/G**	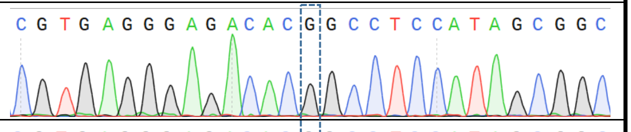
	**Sister**	**Homozygote G/G**	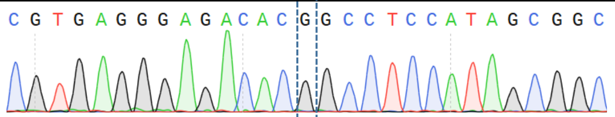
	**Paternal aunt**	**Homozygote G/G**	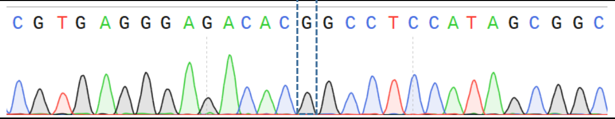

DCHS1 R2462Q rs117140835	**Proband (son)**	**Homozygote G/G**	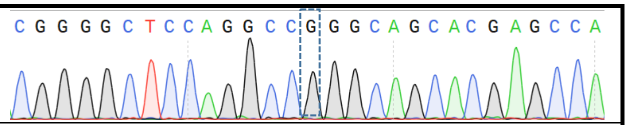
	**Father**	**Homozygote G/G**	Determined by Exome Sequencing
	**Mother**	**Homozygote G/G**	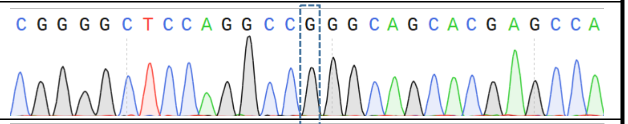
	**Sister**	**Homozygote G/G**	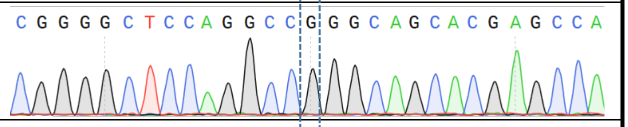
	**Paternal aunt**	**Homozygote G/G**	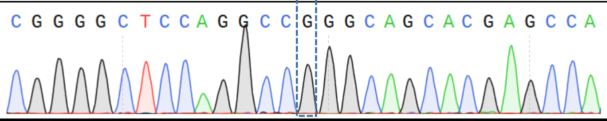

DCHS1 A2464P no rs #	**Proband (son)**	**Homozygote G/G**	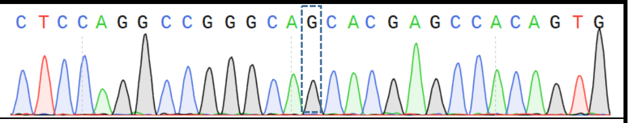
	**Father**	**Homozygote G/G**	Determined by Exome Sequencing
	**Mother**	**Homozygote G/G**	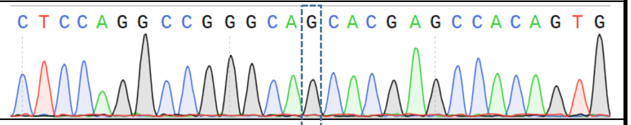
	**Sister**	**Homozygote G/G**	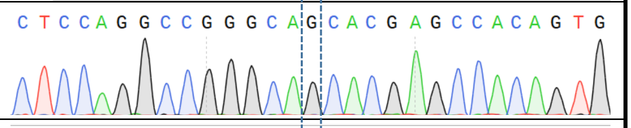
	**Paternal aunt**	**Homozygote G/G**	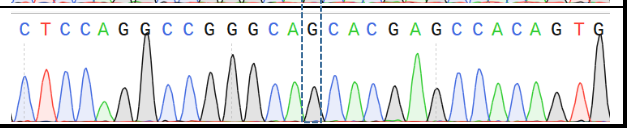

DCHS1 R2513H rs201457110			
	**Proband (son)**	**Homozygote G/G**	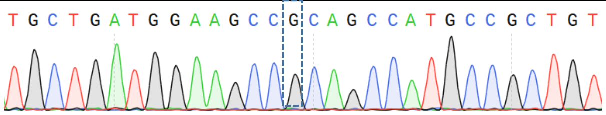
	**Father**	**Homozygote G/G**	Determined by Exome Sequencing
	**Mother**	**Homozygote G/G**	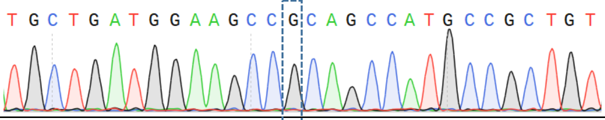
	**Sister**	**Homozygote G/G**	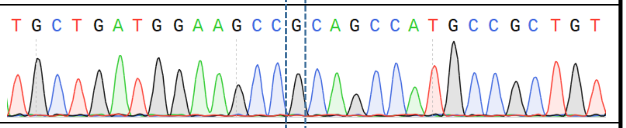
	**Paternal aunt**	**Homozygote G/G**	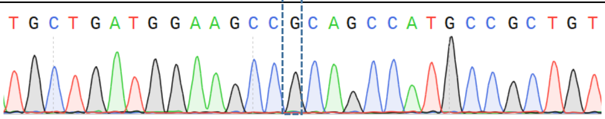

DCHS1 R2770Q rs999967170			
	**Proband (son)**	**Hom ozygote C/C**	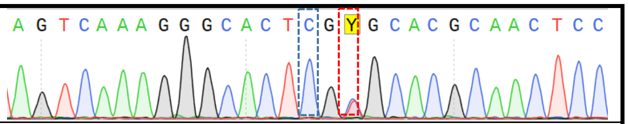
	**Father**	**Homozygote C/C**	Determined by Exome Sequencing
	**Mother**	**Homozygote C/C**	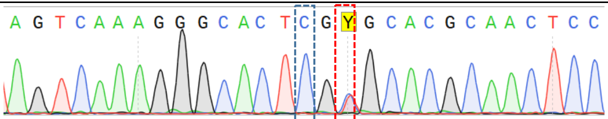
	**Sister**	**Homozygote C/C**	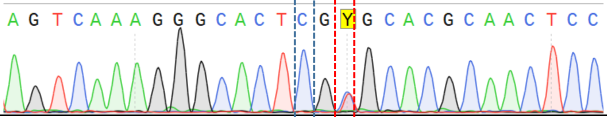
	**Paternal aunt**	**Homozygote C/C**	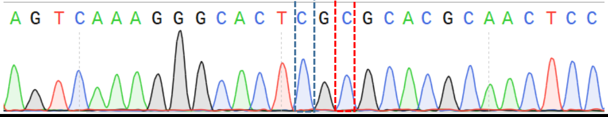

Note: Proband, Mother and Sister are heterozygote C/T for synonymous A2769A, rs72911011. Red box

DCHS1 **R2827P rs35599968**	**Proband (son)**	**Homozygote C/C**	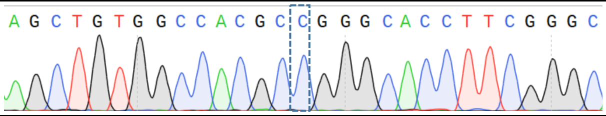
	**Father**	**Homozygote C/C**	Determined by Exome Sequencing
	**Mother**	**Homozygote C/C**	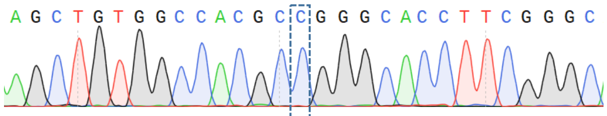
	**Sister**	**Homozygote C/C**	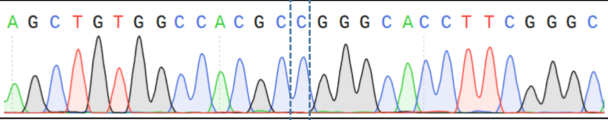
	**Paternal aunt**	**Homozygote C/C**	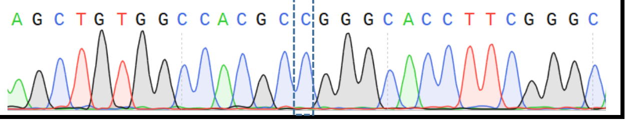

DCHS1 **A2867T rs146233988**	**Proband (son)**	**Homozygote C/C**	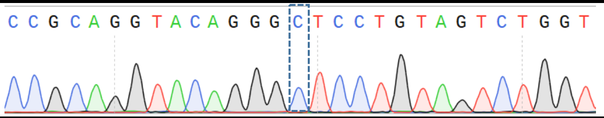
	**Father**	**Homozygote C/C**	Determined by Exome Sequencing
	**Mother**	**Homozygote C/C**	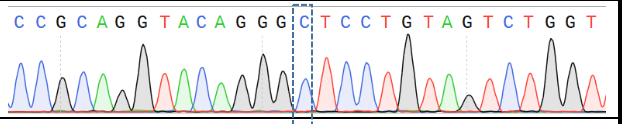
	**Sister**	**Homozygote C/C**	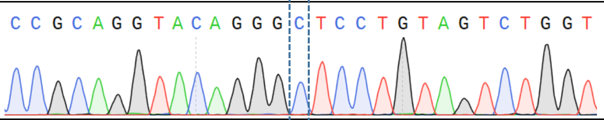
	**Paternal aunt**	**Homozygote C/C**	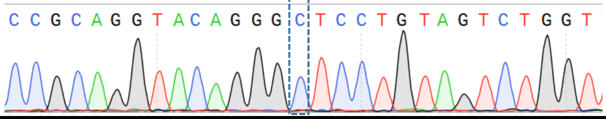

Figure S1. Sanger sequencing results of candidate mutations causing valve diseases in *NOTCH1* (A), *FLNA* (B), and *DCHS1* (C).

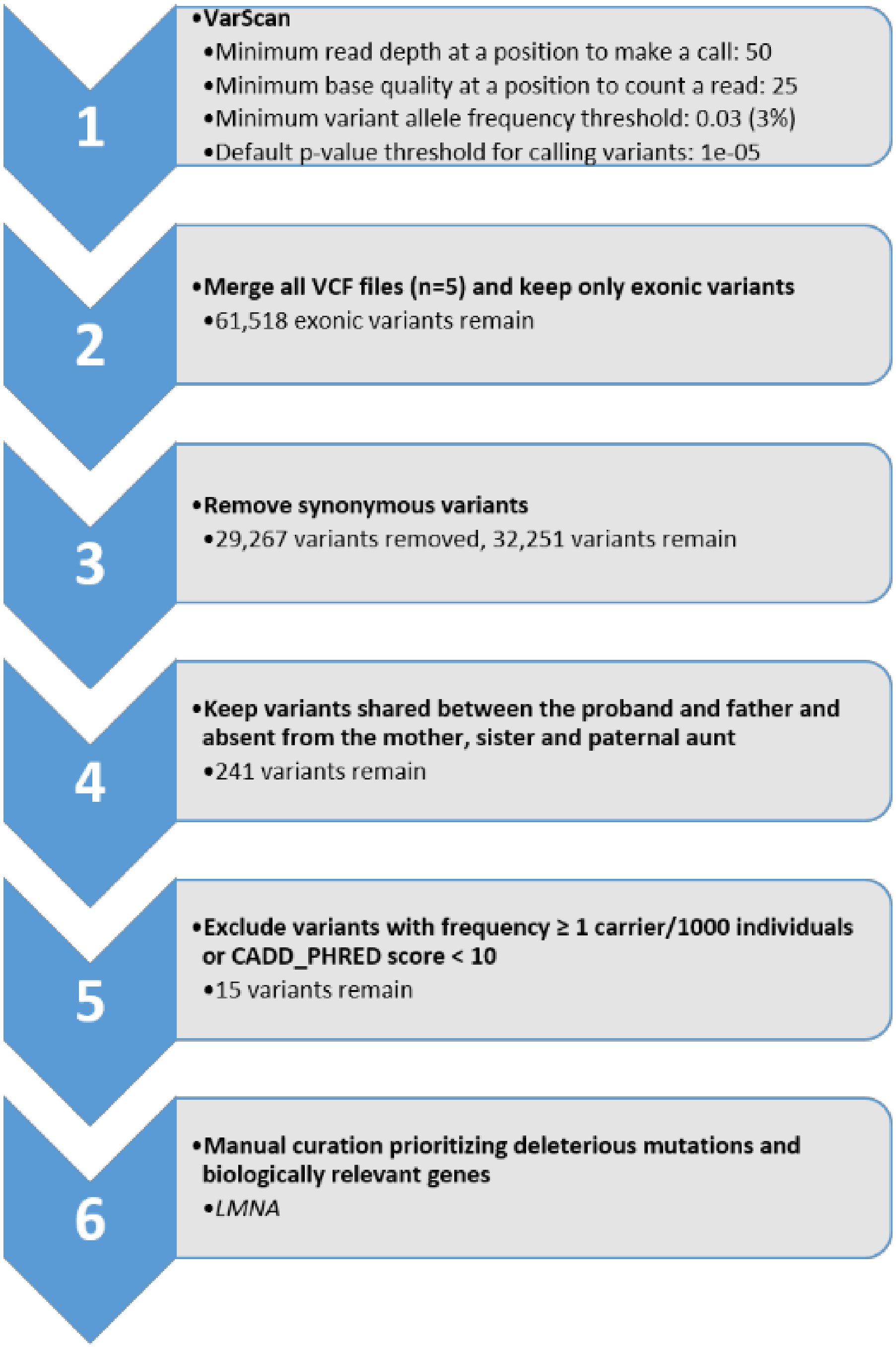

Figure S2. Steps to filter genetic variants identified by whole-exome sequencing.

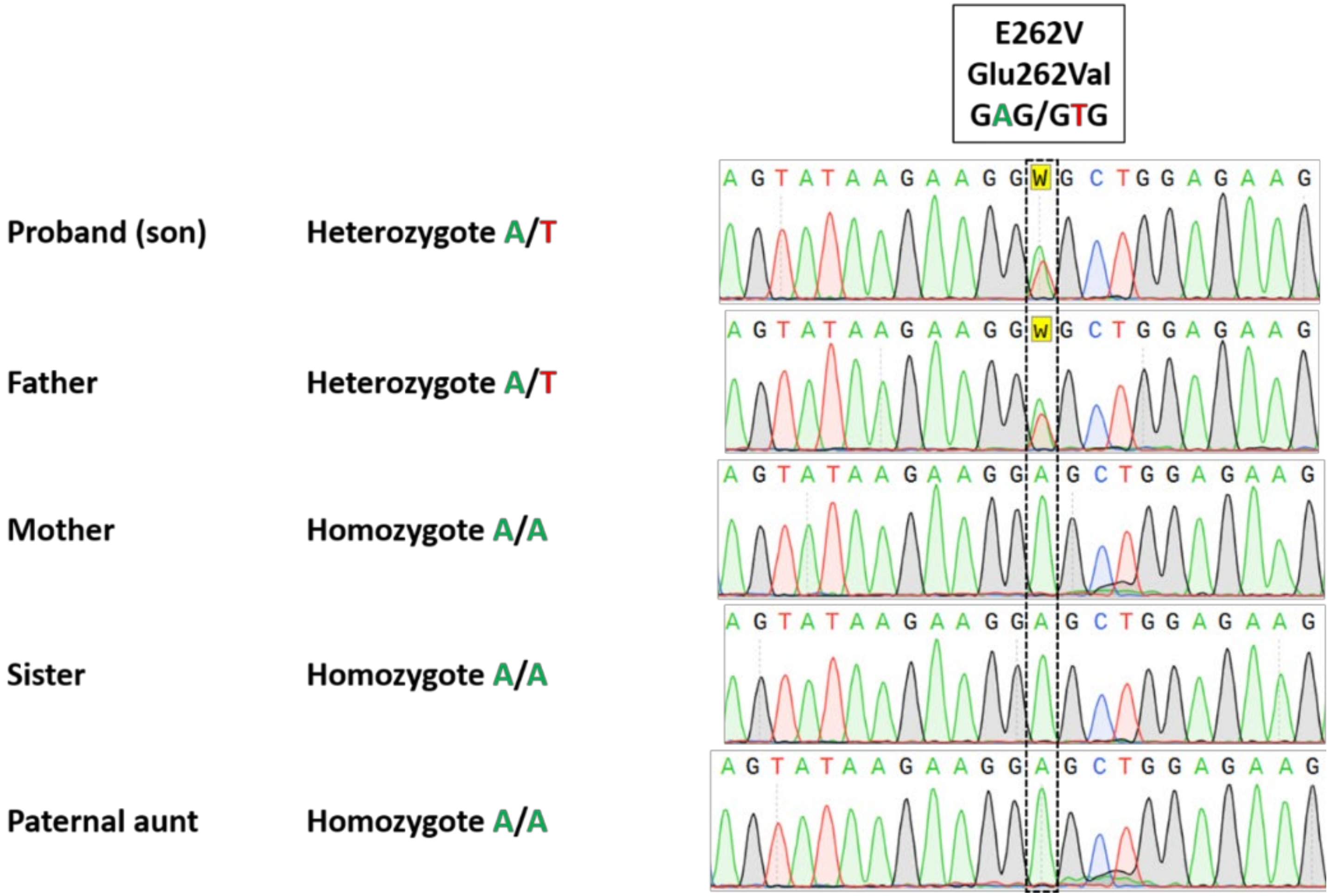

Figure S3. Sanger sequencing confirmation of p.Glu262Val in *LMNA*.

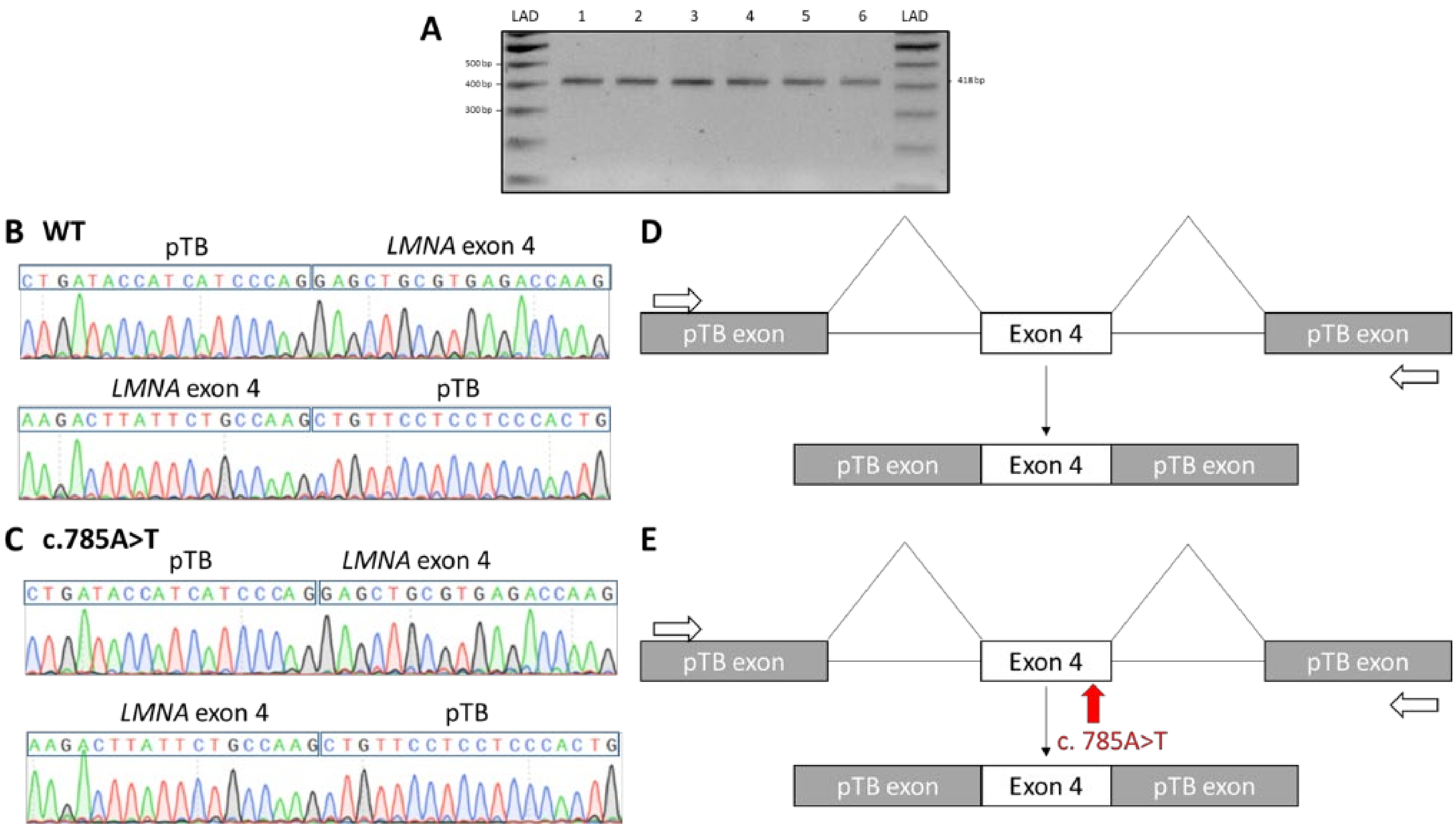

Figure S4. Minigene splicing assay results.

(A) RT-PCR results obtained after transfection of HeLa cells with wild-type (lanes 1 to 3) and mutated (lanes 4 to 6) constructs for *LMNA*: c.785A>T. (B-C) Sanger sequencing results of the product obtained after transfection with wild-type (B) and mutated (C) constructs, using pTB minigene vector. (D-E) Schematic representations of the minigene including *LMNA* exon 4 (white boxes) and intronic regions (black lines) for the wild-type (D) and the mutated (E) constructs.

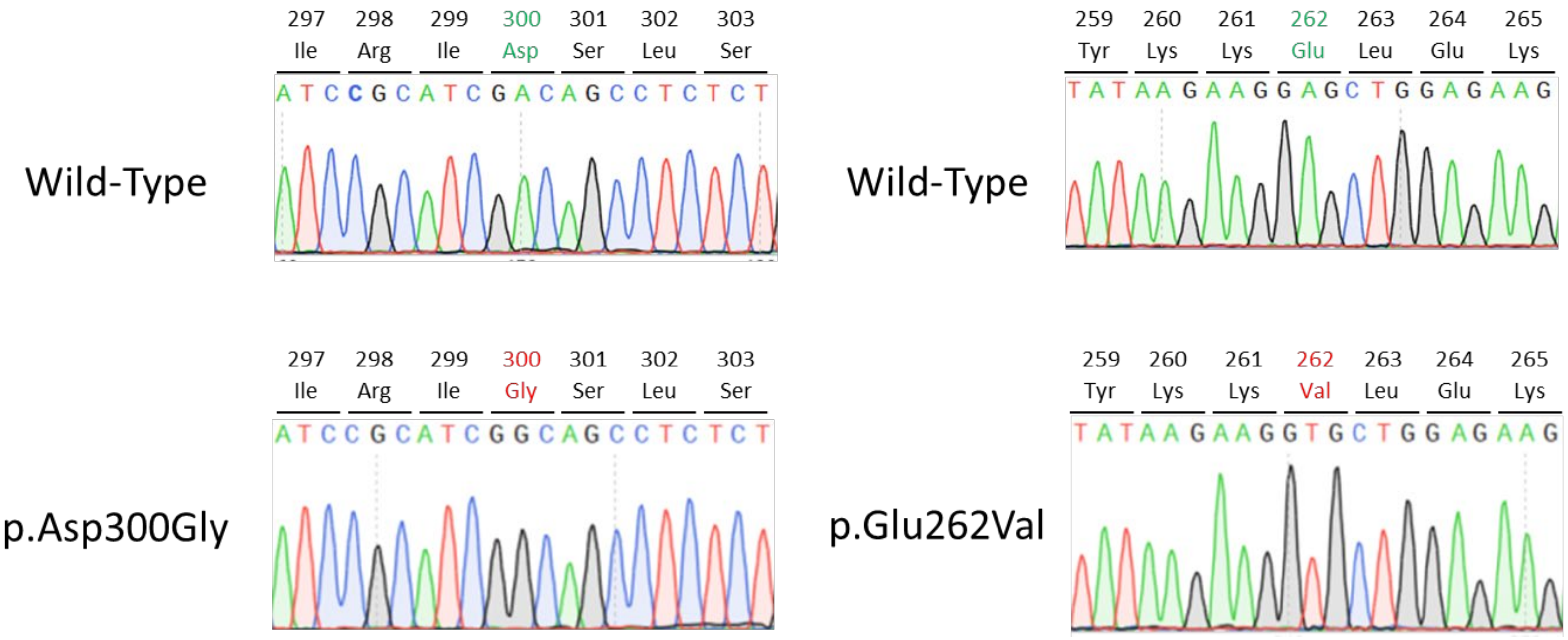

Figure S5. Sanger sequencing results of plasmids.

Nucleotide change	Effect on protein	GnomAD v4		Prediction Algorithms			Domain	Familial Study	Functional study	ACMG criteria for pathogenicity	ACMG	Ref
		n	%	CADD	AlphaMissense	REVEL						
c.4G>A	p.(Glu2Lys)	0	0	24,0	0,85 Likely Pathogenic	0,351 Uncertain	Head	Yes	Yes	PS3, PM1, PM2, PP1	LP	6
c.11C>G	p.(Pro4Arg)	0	0	24,3	0,585 Likely Pathogenic	0,589 Damaging	Head	Yes	Yes	PS3, PP5, PM1, PM5, PP3, PM2	P	7,8
c.29C>T	p.(Thr10Ile)	0	0	23,0	0,356 Ambiguous	0,539 Damaging	Head	Yes	Yes	PS3, PP5, PM1, PM5, PM2	P	8
c.175C>G	p.(Leu59Val)	0	0	26,4	0,978 Likely Pathogenic	0,898 Damaging	Coil 1A	No	No	PM1, PP3, PM5, PM2	LP	7
c.331G>A	p.(Glu111Lys)	0	0	29,4	0,916 Likely Pathogenic	0,779 Damaging	Coil 1B	Yes	Yes	PS3, PM1, PM2, PP5	LP	8
c.398G>T	p.(Arg133Leu)	0	0	25,0	0,775 Likely Pathogenic	0,811 Damaging	Coil 1B	Yes	Yes	PS3, PM5, PP3, PP5, PM1, PM2	P	9,10
c.406G>C	p.(Asp136His)	0	0	28,3	0,943 Likely Pathogenic	0,838 Damaging	Coil 1B	Yes	Yes	PM5, PM1, PP3, PM2	LP	8
c.412G>C	p.(Glu138Gln)	0	0	29,2	0,708 Likely Pathogenic	0,73 Damaging	Coil 1B	Yes	No	PM2, PP3, PP1	VUS	11
c.412G>A	p.(Glu138Lys)	0	0	32,0	0,970 Likely Pathogenic	0,915 Damaging	Coil 1B	Yes	No	PP3, PP5, PM1, PM2	P	12
c.419T>G	p.(Leu140Arg)	0	0	23,5	0,537 Ambiguous	0,702 Damaging	Coil 1B	No	Yes	PS3, PM1, PM5, PM2, PP5	P	13
c.434A>G	p.(Glu145Gly)	0	0	33,0	0,851 Likely Pathogenic	0,764 Damaging	Coil 1B	Yes	No	PM2, PP3, PP1	VUS	11
c.475G>A	p.(Glu159Lys)	0	0	24,7	0,614 Likely Pathogenic	0,773 Damaging	Coil 1B	Yes	Yes	PS3, PP3, PM1, PM2	LP	8
**c.785A>T**	**p.(Glu262Val)**	**0**	**0**	**33,0**	**0,945 Likely Pathogenic**	**0,877 Damaging**	**Coil 2**	**Yes**	**Yes**	**(PP1), (PM1) PS3, PM2, PM5, PP3**	**LP**	**This study**
c.898G>C	p.(Asp300His)	0	0	28,3	0,972 Likely Pathogenic	0,830 Damaging	Coil 2	Yes	No	PM5, PM1, PP3, PM2	LP	14
c.898G>A	p.(Asp300Asn)	0	0	28,8	0,821 Likely Pathogenic	0,606 Damaging	Coil 2	Yes	No	PP5, PM1, PM5, PP3, PM2	LP	15
c.899A>G	p.(Asp300Gly)	0	0	24,4	0,923 Likely Pathogenic	0,833 Damaging	Coil 2	Yes	Yes	PS3, PM1, PM5, PP3, PM2, PP5	P	16

Table S1. List of previously reported *LMNA* variants in patients with valvular phenotype.

Nucleotide change	Effect on protein	Clinical Features	Valvular Features	Premature aging	Metabolic abnormalities	Skin Changes	Neuromuscular Phenotype	Ref
c.4G>A	p.(Glu2Lys)	LCPS	severe calcific aortic stenosis, calcified mitral valve	Yes	No	No	No	6
c.11C>G	p.(Pro4Arg)	APS	Mitral and Aortic Valve Replacement	NR	Partial or Generalized Lipodystrophy	Yes	NR	7,8
c.29C>T	p.(Thr10Ile)	APS	Aortic Stenosis	NR	Diabetes and Generalized Lipodystrophy	Yes	NR	8
c.29C>T	p.(Thr10Ile)	APS	Mitral, Aortic and Tricuspid Regurgitation	NR	Diabetes and Generalized Lipodystrophy	Yes	NR	8
c.175C>G	p.(Leu59Val)	APS	Tricuspid Regurgitation	Yes	Partial Lipodystrophy	Yes	No	17
c.331G>A	p.(Glu111Lys)	APS	Tricuspid Regurgitation	NR	No	Yes	NR	8
c.398G>T	p.(Arg133Leu)	Atypical WS	Thickened valves, aortic regurgitation	Yes	Generalized Lipoatrophy, massive liver steatosis, hypertriglyceridemia, diabetes	Yes	No	9,10
c.406G>C	p.(Asp136His)	APS	Mitral and Aortic Valve Replacement	NR	Diabetes and Partial Lipodystrophy	Yes	NR	8
c.412G>C	p.(Glu138Gln)	Valvular features only	Premature aortic and mitral valve calcification, Severe Aortic Stenosis	No	No	No	No	11
c.412G>A	p.(Glu138Lys)	APS	Mitral, tricuspid and aortic incompetence	Yes	NR	Yes	Yes	12
c.419T>G	p.(Leu140Arg)	Atypical WS	Aortic Stenosis	Yes	No	Yes	NR	13
c.434A>G	p.(Glu145Gly)	Valvular features only	Severe Aortic and mitral valve stenosis and calcifications	No	No	No	No	11
c.475G>A	p.(Glu159Lys)	APS	Mitral and Aortic Valve Replacement	NR	No	Yes	NR	8
**c.785A>T**	**p.(Glu262Val)**	**Valvular features only**	**Severe aortic valve and mitral annulus calcification**	**No**	**No**	**No**	**No**	**This study**
c.898G>C	p.(Asp300His)	Atypical WS	Mitral Calcifications	Yes	Lipoatrophy	Yes	No	14
c.898G>A	p.(Asp300Asn)	Atypical WS	Aortic valve calcifications	Yes	NR	Yes	No	15
c.899A>G	p.(Asp300Gly)	LCPS	Mitral calcification and regurgitation, Aortic valve stenosis	Yes	NR	Yes	NR	16

Table S2. Detailed phenotypes of reported patients with *LMNA* variants associated with valvular abnormalities.

APS	Atypical Progeroid Syndrome
LCPS	*LMNA*-associated cardiocutaneous progeria syndrome
NR	Not Reported
WS	Werner Syndrome

Gene	Variation tested	rs #	Forward	Reverse	PCR *	Amplicon Size (bp)
**PCR and Sanger Sequencing Primers**						
NOTCH1	R1107X	rs41309764	5’-GTCCACCAGGTCCTCACAGT-3’	**5’-TGTGCACTGGTGTGACTCCT-3’**	**A**	357
NOTCH1	H1504del	rs41309766	5’-AAAGGGTTTTGCTGCTGGGG-3’	**5’-CCCCTGAGCAGAGCCTTAGA-3’**	**A**	822
FLNA	G288R	rs267606816	**5’-AACGTGGACGAGCACTCTGTC-3’**	5’-CTCTTGGGTGGCTTGATCACCTAG-3’	**A**	247
FLNA	P637Q<br>V711D	rs267606815<br>rs267606817	5’-TCGCAGGCTAAGATCGAATGTGAC-3’	**5’-GTAGGAGCAGCTGTAAGTGCCATTG-3’**	**B**	603
FLNA	1944del	no rs #	5’-ACCTGCTCCTATCTGCCTGACAG-3’	5’-CCTCAGAGAGCACAGTGGGTTC-3’	**E**	2933 or 989
DCHS1	S415R	rs117368891	5’-CAATGTGTCCCTGGAAGGTGGAGA-3’	5’-CCTGCCAAGACCATCTGCCTC-3’	**C**	815
				**5’-GGAGGTGAGCCTGAGTCTGTG-3’**		Sequencing
DCHS1	R2330C	rs768737101	5’-TCTCCACCCTGCAGCTCAAG-3’	5’-ACTCAGGGAATGGCCTATCTGCT-3’	**C**	1058
				**5’-GTAGTTCACAGGACTTGGGACC-3’**		Sequencing
DCHS1	R2462Q<br>A2464P<br>R2513H<br>R2770Q<br>R2827P<br>A2867T	rs117140835<br>no rs #<br>rs201457110<br>rs999967170<br>rs35599968<br>rs146233988	5’-**AGCAGATAGGCCATTCCCTGAGT**-3’	**5’-CAGCTCCTGGCCCACCATAG-3’**	**D**	1883
				**5’-CATCCTCATCTGTGGCCTGC-3’**		Sequencing
LMNA	E262V	no rs #	5’-CAGCATGAGGACCAGGTGGAG-3’	**5’-CCAGAAGGCATAGCCCAGC-3’**	**C**	140
**Minigene Assay Primers**						
LMNA	EXON 4		5’-CTAAACAGCCACATATGTGTGCAGAGCTCGCCTTC -3’	5’-CCCCCCTCGACCATATGCCTGAGTTGGGCATCACTG -3’	**F**	523
**Site-directed Mutagenesis Primers**						
LMNA	E262V		5’-TATAAGAAGGTGCTGGAGAAGACTTATTCTG -3’	5’- CTGCTCCACCTGGTCCTC -3’		
LMNA	D300G		5’-ATCCGCATCGGCAGCCTCTCTG-3’	5’-GCGCGACTGCTGCAGCTC-3’		

Sequencing primers are shown in bold.
*Refer to Supplementary Table S4 for PCR cycling conditions.

Table S3. Primers to sequence mutations causing valve diseases in the *NOTCH1*, *FLNA* and *DCHS1* genes and to perform site-directed mutagenesis.

PCR condition	Initial Denaturation	Denaturation	Annealing	Elongation
**A**	95°C, 15 min	94°C, 15 sec	57.0°C, 30 sec	72°C, 1 min
**B**	95°C, 15 min	94°C, 15 sec	60.0°C, 30 sec	72°C, 1 min
**C**	95°C, 15 min	94°C, 15 sec	59.0°C, 30 sec	72°C, 1 min
**D**	95°C, 15 min	94°C, 15 sec	59.0°C, 30 sec	72°C, 2 min
**E**	95°C, 15 min	94°C, 15 sec	59.0°C, 30 sec	72°C, 3 min
**F**	98°C, 30 sec	98°C, 10 sec	55.0°C, 30 sec	72°C, 30 sec

Table S4. PCR cycling conditions.

Phenotype	HPO number
Aortic valve calcification	HP:0004380
Mitral annulus calcification	HP:0005136
Aortic valve stenosis	HP:0001650
Mitral valve regurgitation	HP:0001653

Table S5. Human Phenotype Ontology (HPO) annotations of the reported phenotype findings.

Supplemental materials

Samples and DNA extraction

Blood samples were collected in EDTA tubes. After centrifugation, buffy coats were collected and stored at -80°C. DNA from four individuals (proband, mother, sister and paternal aunt) was extracted using the QIAamp DNA Blood Mini kit (Qiagen). DNA from the skin melanoma biopsy (father) was extracted using QIAamp DNA FFPE Tissue kit (Qiagen). The DNA was further purified using Micro Bio-Spin Chromatography-6 Column (Bio-Rad) in order to remove melanin from skin which inhibits polymerase activity. The DNA quality and concentration were assessed by the UV absorbance ratio 260/280 nm and UV absorbance 260 nm, respectively.

Sanger sequencing of candidate genes

Three genes known to cause non-syndromic valve diseases were evaluated, namely *NOTCH1*, *FLNA*, and *DCHS1*. Primers were designed to cover mutations R1107X and H1504del for *NOTCH1*[1]; G288R, P637Q, V711D and 1944bp_del for *FLNA*[2]; and S415R, R2330C, R2462Q, A2464P, R2513H, R2770Q, R2827P and A2867T for *DCHS1*[3,4]. Primer sequences are provided in **Table S3**. PCR was performed in a final volume of 25 µL containing 100 ng of genomic DNA, 1 U of HotStarTaq DNA polymerase (Qiagen), PCR buffer 1X, Q-Solution 1X, 160 µM of each dNTP and 0.2 µM of each primer. The PCR reaction was carried out on the GeneAmp® PCR system 9700 (Applied Biosystems). PCR cycling conditions are provided in **Table S4**. The sequencing reaction was then performed using standard procedures and the product was run on the ABI 3730xl DNA Analyzer (Applied Biosystems). Sequencing files were assembled and analyzed using the EMBL-EBI Clustal Omega Multiple Alignment Tool

(http://www.ebi.ac.uk/Tools/msa/clustalo). For the deletion of 1944 bp in *FLNA*, the DNA fragment was amplified and separated by electrophoresis using 1% agarose gel (**Figure S1**).

Minigene Splicing Reporter Assay

Minigene Splicing reporter assay was performed as previously described [5]. Briefly, a genomic region spanning exon 4 of *LMNA* (227bp in intron 3 and 154bp in intron 4) was amplified from the proband DNA using the Q5® High-Fidelity DNA Polymerase (New England Biolabs) according to the manufacturer`s instructions. The primers used are described in **Table S3**. Amplicons were inserted in the *NdeI* restriction site of the previously described pTB minigene vector using the In-fusion Snap Assembly cloning kit (Takara Bio). DNA sequences of wild-type and mutant plasmids were verified by Sanger sequencing. HeLa cells were plated at a concentration of 5×10^4 cells/well in a 12-well cluster plate in 1ml of growth medium and transiently transfected with 1 μg of plasmid using JetOPTIMUS® (PolyPlus) one day after seeding following the manufacturer's recommendations. Forty-eight hours after transfection, total RNA was extracted from the cells using RNeasy Plus Micro Kit (Qiagen). RT-PCR (reverse transcription polymerase chain reaction) was performed with Quantitect Reverse Transcription Kit (Qiagen) using 250 ng of total RNA and random primers. Complementary DNA (cDNA) amplification was performed using vector-specific primers surrounding the cloning site and HotStar Taq Plus DNA polymerase (Qiagen). The PCR products were resolved on a 2% agarose gel and Sanger sequenced to identify splicing events. All transfection experiments were performed in triplicate.

Construction of vectors

The cDNA constructs harboring the E262V and D300G mutations were verified by Sanger sequencing (**Figure S5**) and primers are listed in **Table S3**.

Supplemental References

1. Garg, V., Muth, A.N., Ransom, J.F., Schluterman, M.K., Barnes, R., King, I.N., Grossfeld, P.D., and Srivastava, D. (2005). Mutations in NOTCH1 cause aortic valve disease. Nature *437*, 270–274. https://doi.org/10.1038/nature03940.

2. Kyndt, F., Gueffet, J.-P., Probst, V., Jaafar, P., Legendre, A., Le Bouffant, F., Toquet, C., Roy, E., McGregor, L., Lynch, S.A., et al. (2007). Mutations in the gene encoding filamin A as a cause for familial cardiac valvular dystrophy. Circulation *115*, 40–49. https://doi.org/10.1161/CIRCULATIONAHA.106.622621.

3. Durst, R., Sauls, K., Peal, D.S., deVlaming, A., Toomer, K., Leyne, M., Salani, M., Talkowski, M.E., Brand, H., Perrocheau, M., et al. (2015). Mutations in DCHS1 cause mitral valve prolapse. Nature *525*, 109–113. https://doi.org/10.1038/nature14670.

4. Clemenceau, A., Bérubé, J.-C., Bélanger, P., Gaudreault, N., Lamontagne, M., Toubal, O., Clavel, M.-A., Capoulade, R., Mathieu, P., Pibarot, P., et al. (2018). Deleterious variants in DCHS1 are prevalent in sporadic cases of mitral valve prolapse. Mol. Genet. Genomic Med. *6*, 114–120. https://doi.org/10.1002/mgg3.347.

5. Janin, A., Chanavat, V., Rollat-Farnier, P.-A., Bardel, C., Nguyen, K., Chevalier, P., Eicher, J.-C., Faivre, L., Piard, J., Albert, E., et al. (2020). Whole MYBPC3 NGS sequencing as a molecular strategy to improve the efficiency of molecular diagnosis of patients with hypertrophic cardiomyopathy. Hum. Mutat. *41*, 465–475. https://doi.org/10.1002/humu.23944.

6. Wilke, M.V.M.B., Wick, M., Schwab, T.L., Starosta, R.T., Clark, K.J., Connolly, H.M., and Klee, E.W. (2024). Nuclear Abnormalities in LMNA p.(Glu2Lys) Variant Segregating with LMNA-Associated Cardiocutaneous Progeria Syndrome. Genes *15*, 112. https://doi.org/10.3390/genes15010112.

7. Guo, H., Luo, N., Hao, F., and Bai, Y. (2014). p.Pro4Arg mutation in LMNA gene: a new atypical progeria phenotype without metabolism abnormalities. Gene *546*, 35–39. https://doi.org/10.1016/j.gene.2014.05.042.

8. Garg, A., Subramanyam, L., Agarwal, A.K., Simha, V., Levine, B., D'Apice, M.R., Novelli, G., and Crow, Y. (2009). Atypical progeroid syndrome due to heterozygous missense LMNA mutations. J. Clin. Endocrinol. Metab. *94*, 4971–4983. https://doi.org/10.1210/jc.2009-0472.

9. Caux, F., Dubosclard, E., Lascols, O., Buendia, B., Chazouillères, O., Cohen, A., Courvalin, J.-C., Laroche, L., Capeau, J., Vigouroux, C., et al. (2003). A new clinical condition linked to a novel mutation in lamins A and C with generalized lipoatrophy, insulin-resistant diabetes, disseminated leukomelanodermic papules, liver steatosis, and cardiomyopathy. J. Clin. Endocrinol. Metab. *88*, 1006–1013. https://doi.org/10.1210/jc.2002-021506.

10. Jacob, K.N., Baptista, F., dos Santos, H.G., Oshima, J., Agarwal, A.K., and Garg, A. (2005). Phenotypic heterogeneity in body fat distribution in patients with atypical Werner's

syndrome due to heterozygous Arg133Leu lamin A/C mutation. J. Clin. Endocrinol. Metab. *90*, 6699–6706. https://doi.org/10.1210/jc.2005-0939.

11. Wu, H.W., Van De Peppel, I.P., Rutten, J.W., Jukema, J.W., Aten, E., Jazet, I.M., Koopmann, T.T., Barge-Schaapveld, D.Q.C.M., and Ajmone Marsan, N. (2024). Atypical Progeria Primarily Manifesting as Premature Cardiac Valvular Disease Segregates with LMNA-Gene Variants. J. Cardiovasc. Dev. Dis. *11*, 86. https://doi.org/10.3390/jcdd11030086.

12. Doubaj, Y., De Sandre-Giovannoli, A., Vera, E., Navarro, C.L., Elalaoui, S.C., Tajir, M., Lévy, N., and Sefiani, A. (2012). An inherited *LMNA* gene mutation in atypical Progeria syndrome. Am. J. Med. Genet. A. *158A*, 2881–2887. https://doi.org/10.1002/ajmg.a.35557.

13. Chen, L., Lee, L., Kudlow, B.A., Dos Santos, H.G., Sletvold, O., Shafeghati, Y., Botha, E.G., Garg, A., Hanson, N.B., Martin, G.M., et al. (2003). LMNA mutations in atypical Werner's syndrome. The Lancet *362*, 440–445. https://doi.org/10.1016/S0140-6736(03)14069-X.

14. Yanhua, X., and Suxian, Z. (2018). Cerebral Haemorrhage in a Young Patient With Atypical Werner Syndrome Due to Mutations in LMNA. Front. Endocrinol. *9*, 433. https://doi.org/10.3389/fendo.2018.00433.

15. Renard, D., Fourcade, G., Milhaud, D., Bessis, D., Esteves-Vieira, V., Boyer, A., Roll, P., Bourgeois, P., Levy, N., and De Sandre-Giovannoli, A. (2009). Novel *LMNA* Mutation in Atypical Werner Syndrome Presenting With Ischemic Disease. Stroke *40*. https://doi.org/10.1161/STROKEAHA.108.531780.

16. Kane, M.S., Lindsay, M.E., Judge, D.P., Barrowman, J., Ap Rhys, C., Simonson, L., Dietz, H.C., and Michaelis, S. (2013). LMNA-associated cardiocutaneous progeria: an inherited autosomal dominant premature aging syndrome with late onset. Am. J. Med. Genet. A. *161A*, 1599–1611. https://doi.org/10.1002/ajmg.a.35971.

17. Guo, X., Ling, C., Liu, Y., Zhang, X., and Zhang, S. (2016). A Case of Novel Lamin A/C Mutation Manifesting as Atypical Progeroid Syndrome and Cardiomyopathy. Can. J. Cardiol. *32*, 1166.e29-31. https://doi.org/10.1016/j.cjca.2015.11.011.
