# Supplementary material for: Early onset multivalvular disease caused by a missense variant in lamin A/C
Source: HGG Adv. 2025 Aug 8;6(4):100491. doi: 10.1016/j.xhgg.2025.100491 (PMC12398237; doi:10.1016/j.xhgg.2025.100491)
Supplement: Document S2. Article plus supplemental information [file mmc2.pdf]

HGG
Advances

BRIEF COMMUNICATION

Early onset multivalvular disease caused by a missense variant in lamin A/C

Alexandre Janin,[1,2] Nathalie Gaudreault,[1] Victoria Saavedra Armero,[1] Zhonglin Li,[1] Ran Xu,[1] Dominique K. Boudreau,[1] Lily Frenette,[1] Julien Ternacle,[1] Danielle Tardif,[1] Sébastien Thériault,[1] Philippe Pibarot,[1] Patrick Mathieu,[1] Christian Steinberg,[1] and Yohan Bossé[1,3,4,*]

Summary

Lamins A/C, coded by *LMNA* gene, are crucial for nuclear architecture preservation. Pathogenic *LMNA* variants cause a wide range of inherited diseases called "laminopathies". A subgroup is referred to "progeroid syndromes" characterized by premature aging and other manifestations including cardiac valve abnormalities. Atypical phenotypes, generally less severe, have also been reported. We report the case of a 26-year-old male with calcific tricuspid aortic and mitral valve diseases. His father was diagnosed with severe aortic valve stenosis and mitral annulus calcification at the age of 38. The goal of this study was to identify the putative variant causing this non-syndromic multivalvular disease. Known disease-causing variants in *NOTCH1*, *FLNA*, and *DCHS1* were first excluded by Sanger sequencing. Whole-exome sequencing was then performed in five family members. A *LMNA* variant (p.Glu262Val) was identified with *in silico* evidences of pathogenicity (CADD [combined annotation dependent depletion] = 33). Cells transfected with the cDNA construct harboring p.Glu262Val were characterized by abnormal nuclear morphology. Along with a literature review, the variant was classified as likely pathogenic. Elucidating the mechanism by which *LMNA* p.Glu262Val specifically affects cardiac heart valves is likely to provide insight about the pathogenesis of Mendelian forms of valvular heart diseases and may help guide the development of therapies.

Introduction

Population-based genomic approaches are starting to elucidate the genetic component of valvular heart diseases (VHD).[1–3] There are however only a few non-syndromic genes known to cause Mendelian forms of VHD including notch receptor 1 (*NOTCH1*) associated with developmental valve anomalies and severe valve calcification,[4] filamin A (*FLNA*) associated with myxomatous valvular dystrophy,[5] and dachsous cadherin-related 1 (*DCHS1*) associated with mitral valve prolapse.[6] These genes were identified by the presence of more than one variant known to cause the disease and further investigations in other families and sporadic cases have revealed additional likely pathogenic variants.[7,8] Screening for variants in these three genes is thus a prerequisite in genetically suspected cases of early onset valve disease.

In this study, we evaluated a single family characterized by premature multivalvular disease. A Mendelian autosomal-dominant inheritance was suspected, but the affected cases were free of known disease-causing variants in *NOTCH1*, *FLNA*, and *DCHS1*. The goal of this study was to identify the putative causal variant and gene.

Material and methods

Ethics statement

All individuals provided written informed consent, and the ethics committee of the Institut universitaire de cardiologie et de pneumologie de Québec – Université Laval approved the study (no. 20341).

DNA and clinical assessment

Three family members visited our research institution for an echocardiogram and genetic testing. The paternal aunt sent a blood sample and echocardiogram report from another institution. Blood from the deceased father was not available; however, a formalin-fixed paraffin-embedded skin melanoma biopsy was shipped to our laboratory. Sample processing and DNA extraction are provided in the supplemental information.

WES

Libraries were generated using the SureSelectXT Low Input Automated Target Enrichment for Illumina Paired-End Multiplexed Sequencing (Agilent) and using SureSelect Human All Exon V7 as per the manufacturer's recommendations. Captured libraries were quantified using the Kapa Illumina GA with Revised Primers-SYBR Fast Universal kit (Kapa Biosystems). Average size fragment was determined using a LabChip GX (PerkinElmer)

[1]Institut universitaire de cardiologie et de pneumologie de Québec – Université Laval, Quebec City, QC G1V 4G5, Canada; [2]Université Claude Bernard Lyon 1, Université de Lyon, Lyon 69008, France; [3]Department of Molecular Medicine, Université Laval, Quebec City, QC G1V 0A6, Canada
[4]Lead contact
*Correspondence: yohan.bosse@criucpq.ulaval.ca
https://doi.org/10.1016/j.xhgg.2025.100491.
© 2025 The Author(s). Published by Elsevier Inc. on behalf of American Society of Human Genetics.
This is an open access article under the CC BY license (http://creativecommons.org/licenses/by/4.0/).

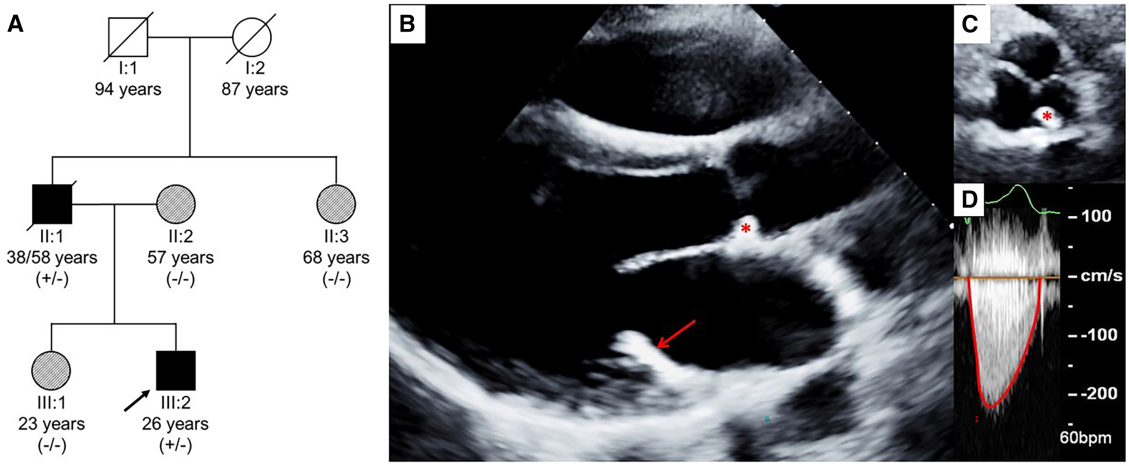

Figure 1. Family pedigree and phenotype
(A) Family pedigree. Each symbol indicates a female (circle) or male (square) family member. Black symbols indicate affected individuals, gray ones indicate unaffected family members and white ones indicate unphenotyped relatives. The text immediately below the symbols gives the family member ID; age at the echocardiogram evaluation or age of death when applicable (presented as age of diagnosis/age of death); and the *LMNA* Glu262Val variant status. A diagonal line across symbol indicates that the individual is deceased. The index case (black arrow) of this family is individual III:2.
(B–D) Two-dimensional echocardiography of the proband. (B) Parasternal long-axis view. (C) Parasternal short-axis view of the aortic valve. (D) Transaortic valvular flow velocity measured by continuous-wave Doppler ultrasound. Red star: calcification of the base of the left-coronary aortic valve cusp. Red arrow: thickening and calcification of the posterior mitral valve leaflet.

instrument. DNA sequencing was performed on the Illumina NovaSeq 6000 S4 flow cell.

In silico filtering of the putative variant

Allele frequencies of identified genetic variants were compared with publicly available databases including the Genome Aggregation Database (gnomAD version 4),[9] and the variant browser Bravo from the NHLBI's TOPMed program (https://bravo.sph.umich.edu/). Pathogenicity of missense variants was evaluated using PolyPhen,[10] AlphaMissense,[11] and the CADD framework.[12] To evaluate the effect on splicing, two algorithms were used: MaxEntScan[13] and SpliceAI.[14] The ACMG (American College of Medical Genetics and Genomics) guidelines were used for the interpretation of variants.[15]

Vectors construction, transfection, and nuclear morphology assessment

The expression vector containing the wild-type (WT) *LMNA* cDNA (NM_170707) sequence fused to green fluorescent protein (GFP) was purchased from SinoBiological (Ref HG12058-ACG, Vector backbone pCMV3-C-GFPSpark). E262V and D300G plasmids were generated by site-directed mutagenesis using the Q5 Site-Directed Mutagenesis Kit (New England Biolabs). Sanger sequencing of plasmids and primers are in supplemental information.

HEK293T cells were seeded at 1.3×10^5 cells/well in a 6-well plate with 2 mL growth medium the day before transfection. Each plasmid (2 μg) was transfected using Lipofectamine 3000 (Invitrogen) as per the manufacturer's instructions. After 72 h, cells were passed into medium containing 25 μg/mL hygromycin B (Sigma-Aldrich) for selection. Approximately 2 weeks later, single colonies were isolated and then expanded to make stable cell lines.

Cells were fixed with 4% paraformaldehyde for 10 min, washed with PBS, permeabilized in PBS with 0.1% Triton X-100 for 30 min, and blocked in PBS containing 1% BSA and 10% FBS for 30 min. Nuclei were stained with DAPI for 30 min. Immunofluorescent images were acquired with a Zeiss LSM800 Axio Observer Z1 microscope (ZEN software, version 2.3) and analyzed with ImageJ (version 1.54). Nuclear shape parameters (area, perimeter, and nuclear contour ratio) were estimated by ImageJ "Analyze Particles" plugin with default parameters (version 1.54). Juxtaposed or overlapping nuclei were manually corrected. Automatic thresholding of the 8-bit images was performed. Parameters included a minimum size of 10 pixels, circularity of 0.1–1.0, and exclusion of edge-touching particles. The nuclear contour ratio, defined as $4\pi A/P^2$, where A is the nuclear area and P is the perimeter, was calculated to assess circularity (1 = perfect circle). Results are expressed as mean ± standard deviation.

Results

Family history

A family was referred to us with non-syndromic early onset multivalvular disease. The mother reported that her husband had severe aortic valve and mitral annulus calcification diagnosed at the age of 38. He had succumbed from the disease at 58 after several cardiac surgeries. The severe multivalvular phenotype at this young age remained unexplained. After her husband passed away, her 26-year-old son (proband) and a 23-year-old daughter, underwent an echocardiography screening. He had normal appearance with no sign of premature aging, skin disease, or dysmorphic feature. However, the transthoracic echocardiography revealed isolated mild aortic valve stenosis (aortic valve area of 1.39 cm^2, transvalvular peak velocity of 2.1 m/s, and transvalvular peak and mean gradients of 18 and 11 mmHg, respectively) and mild mitral valve regurgitation likely related to leaflet thickening. The thickness of the anterior mitral leaflet was 4.7 mm (Figure 1). The left

ventricular ejection fraction (56%) and left ventricular end diastolic diameter (50.1 mm) were preserved. The echocardiography findings mirrored the phenotype documented in his father's medical record. The proband's mother, sister, and paternal aunt had normal echocardiogram. None of the family members displayed features of a dilated cardiomyopathy or atrioventricular conduction defects. The clinical presentation suggested an autosomal-dominant inheritance. A genetic investigation was thus initiated to identify the molecular cause of valve disease. Accordingly, the son in this report is considered the proband (Figure 1). A two-step strategy was used to identify the genetic etiology, first testing candidate genes and then whole-exome sequencing (WES).

Genetic testing

Genetic testing excluded known variants causing valve diseases in *NOTCH1*, *FLNA*, and *DCHS1* (see supplemental information and Figure S1). WES was performed in the five family members. Steps to call and filter genetic variants are depicted in Figure S2. Briefly, by combining the VCF files of the five individuals, 61,518 exonic variants were identified. After the exclusion of synonymous variants, we kept variants shared between the proband and the father and absent from the mother, sister, and paternal aunt. The 241 remaining variants were further filtered based on minor allele frequencies of <0.1% in publicly available databases and a pathogenicity CADD score of at least 10. The 15 remaining variants are indicated in Table 1. Among them, 2 are not represented in gnomAD version 4. The variant located in *MRM3* is predicted to be benign based on PolyPhen and AlphaMissense, whereas the variant in *LMNA* is predicted to be damaging by pathogenicity scores (Table 1). Taking together, the evidence suggests that the candidate pathogenic variant is in the *LMNA* gene, which we prioritized in downstream follow-up experiments.

In silico evaluation of the *LMNA* variation

The proband and the father are carriers of a missense variant in *LMNA* (Figure 2A). This variant in exon 4 (NM_170707.4:c.785A>T, p.Glu262Val) replaces glutamic acid (acidic) with valine (nonpolar) at codon 262. It has not been reported in the ClinVar database and is predicted to be damaging by PolyPhen2 (0.987), CADD (33.0), AlphaMissense (0.945), and Rare Exome Variant Ensemble Learner (0.877) algorithms (Tables 1 and S1). The Glu262 residue, located in the coil 2 domain (Figure 2B), is highly conserved across species (Figure 2C). A variation affecting the same amino acid (p.Glu262Lys) has been reported as "likely pathogenic" in the ClinVar database and associated with a *LMNA*-related phenotype (Hutchinson-Gilford progeria syndrome, ID: 1698456). Sanger sequencing (Figure S3) confirmed the exome sequencing result.

Additionally, *in silico* analysis showed that the substitution created a relatively strong donor splice site in exon 4. According to MaxEntScan, the *de novo* donor splice site shows a close strength (4.0) to the natural donor splice site (4.7). SpliceAI algorithms confirmed the potential creation of *de novo* donor splice site (donor gain = 0.91).

Based on these *in silico* data, and given a low rate of benign missense variations in *LMNA*, the variant should be considered likely pathogenic according to the ACMG classification (PP2, PM2, PM5, and PP3).[15] *In vitro* studies were undertaken to assess effects on splicing and nuclear architecture to better validate pathogenicity.

Functional assays

Minigene reporting splicing assays demonstrated that p.Glu262Val was not associated with any splicing effects in our experimental workflow (see supplemental information and Figure S4). In contrast, the fluorescence analysis of nuclei from HEK293T transfected with LMNA-GFP plasmids showed abnormal morphology, as previously described for the p.Asp300Gly variant, used as positive control[16,17] (Figure 3A). Images were analyzed to determine three nuclear parameters: area, perimeter, and nuclear contour ratio. We evaluated 90 nuclei for the WT condition, 76 for the p.Asp300Gly variant, and 94 for the p.Glu262Val variant.

Relative to the WT transfected cells, p.Asp300Gly and p.Glu262Val nuclei had significantly smaller nuclear areas than controls (Figure 3B, 129.9 ± 28.1, $p < 0.0001$ for p.Asp300Gly and 203.9 ± 49.7, $p = 0.009$ for p.Glu262Val versus 224.4 ± 29.7 for WT). Although nuclei perimeters were not significantly different for p.Glu262Val (55.9 ± 6.5, $p = 0.053$) compared with WT (57.4 ± 4.0), it was the case for p.Asp300Gly (47.5 ± 6.9, $p < 0.0001$) (Figure 3C). Finally, significant differences were found in the nuclear contour ratio between p.Asp300Gly and WT (0.73 ± 0.09 versus 0.85 ± 0.05, $p < 0.0001$) and between p.Glu262Val and WT (0.81 ± 0.06 versus 0.85 ± 0.05, $p < 0.0001$) (Figure 3D). The area and perimeter for the positive control variant were as reported previously.[18] Based on the results of *in vitro* assays, the PS3 argument of the ACMG classification could be added.[15]

Previously reported *LMNA* variants in valvular diseases

To date, 15 *LMNA* variants have been associated with a valvular phenotype in the literature and in the HGMD database (Tables S1 and S2). The variants reported in classical Hutchinson-Gilford progeria syndrome (HGPS) and Werner syndrome (WS) were excluded, given the more severe phenotype of these diseases. All of these are missense variants in a heterozygous state. All are also absent in gnomAD database (version 4) and the majority (13/15) are considered as likely pathogenic or "pathogenic" according to ACMG guidelines. These variants are spread all over the gene. Only six were found in patients with valvular calcifications (Table S2). Two variants (p.Glu138Gln and p.Glu145Gly) are associated with non-syndromic valvular features and no other abnormalities such as premature aging, skin changes,

Table 1. List of candidate variants from the whole-exome sequencing

Variation	Consequence	Gene [OMIM]	Existing variation	gnomAD NFE AlleleFreq	gnomAD allele count and AlleleFreq	TOPMed (allele count)	CADD PHRED v.1.7	PolyPhen HVAR Score and prediction	AlphaMissense Score and class
1_52810458_G/A	missense	*ZFYVE9* [603755]	rs140709371	0.0002405	234, 0.0008291	148<br>2 HMZ	28.5	0.704 possibly damaging	0.085 likely benign
1_153916583_C/T	missense	*DENND4B* [619843]	rs200116588	0.00002432	48, 0.0001774	119<br>no HMZ	21.3	0.008 benign	0.082 likely benign
**1_156104741_A/T**	**missense**	***LMNA*** [150330]	–	–	–	**0**	33	**0.987 probably damaging**	**0.945 likely pathogenic**
1_161987291_T/C	missense	*OLFML2B*	rs145092080	0.0002971	193, 0.0006945	174<br>no HMZ	20.4	0.294 benign	0.076 likely benign
2_60773406_C/G	missense	*BCL11A* [606557]	rs150125298	0.0003891	160, 0.0005671	134<br>no HMZ	24.3	0.247 benign	0.191 likely benign
5_68616225_G/A	missense	*CCDC125* [613781]	rs111417600	0.00003516	76, 0.0003022	42<br>no HMZ	24.0	0.962 probably damaging	0.123 likely benign
7_38803086_A/G	missense	*VPS41* [605485]	rs148532572	0.001148	202, 0.0007250	189<br>no HMZ	23.6	0.033 benign	0.698 likely pathogenic
10_72511880_C/T	missense	*ADAMTS14* [607506]	rs142585866	0.0002828	234, 0.0008354	290<br>no HMZ	34	0.932 probably damaging	0.52 ambiguous
11_5529215_C/T	missense	*UBQLN3* [605473]	rs147771363	0.0004595	308, 0.001093	433<br>3 HMZ	21.6	0.02 benign	0.067 likely benign
14_19378000_A/G	missense	*OR11H12*	rs761048370	0.0007870	187, 0.0008659	2366<br>0 HMZ	22.4	0.995 probably damaging	0.176 likely benign
16_22358755_G/A	missense	*CDR2* [117340]	rs372728039	0.00001556	14, 0.00004968	12<br>no HMZ	16.51	0.007 benign	0.094 likely benign
16_22545898_A/C	missense	*NPIPB5*	rs774565848	0.000007964	19, 0.00007549	439<br>1 HMZ	8.709	0.923 probably damaging	0.45 ambiguous
17_686469_A/C	missense	*MRM3, RNMTL1*	–	–	–	0	23.4	0.262 benign	0.144 likely benign
18_29788170_G/A	stop gained	*MEP1B* [600389]	rs200474192	0.0001402	179, 0.0006378	151<br>1 HMZ	42	–	–
20_62221499_C/A	missense	*GMEB2* [607451]	rs756634351	0.00002987	13, 0.00005956	14<br>no HMZ	19.21	0.006 benign	0.084 likely benign

The *LMNA* variant identified in this study is in bold.

neuromuscular, or metabolic phenotypes.[17] These are located in the coil 1A region, whereas the reported variant is located at the beginning of the coil 2 domain (Table S1).

Discussion

The indication for genetic testing in this study was a familial form of early onset aortic valve and mitral annulus calcification that was likely transmitted in an autosomal-dominant mode. After ruling out variants known to cause non-syndromic valve disease in *NOTCH1*, *FLNA*, and *DCHS1*, WES was performed. We identified a missense variant, p.Glu262Val, in *LMNA* with prediction of damaging effect by *in silico* testing.

LMNA encodes the A-type nuclear lamins (lamin A and lamin C), intermediate filament proteins essential for the nuclear envelope's structural integrity. Hundreds of *LMNA* variants cause laminopathies, which manifest in diverse phenotypes, including premature aging and different forms of cardiomyopathy, muscular dystrophy, lipodystrophy, and neurodegenerative disorders.[19] Interestingly, cardiac valve calcification and dysfunction are clinical features observed in HGPS, WS, and atypical progeroid syndromes, which exhibit milder phenotypes compared with classic forms.[20] Furthermore, two variants (p.Glu138Gln and p.Glu145Gly) have been associated with non-syndromic valvular abnormalities.[17] This study emphasizes the well-known heterogeneity of laminopathies.[19] Moreover, deciphering the molecular

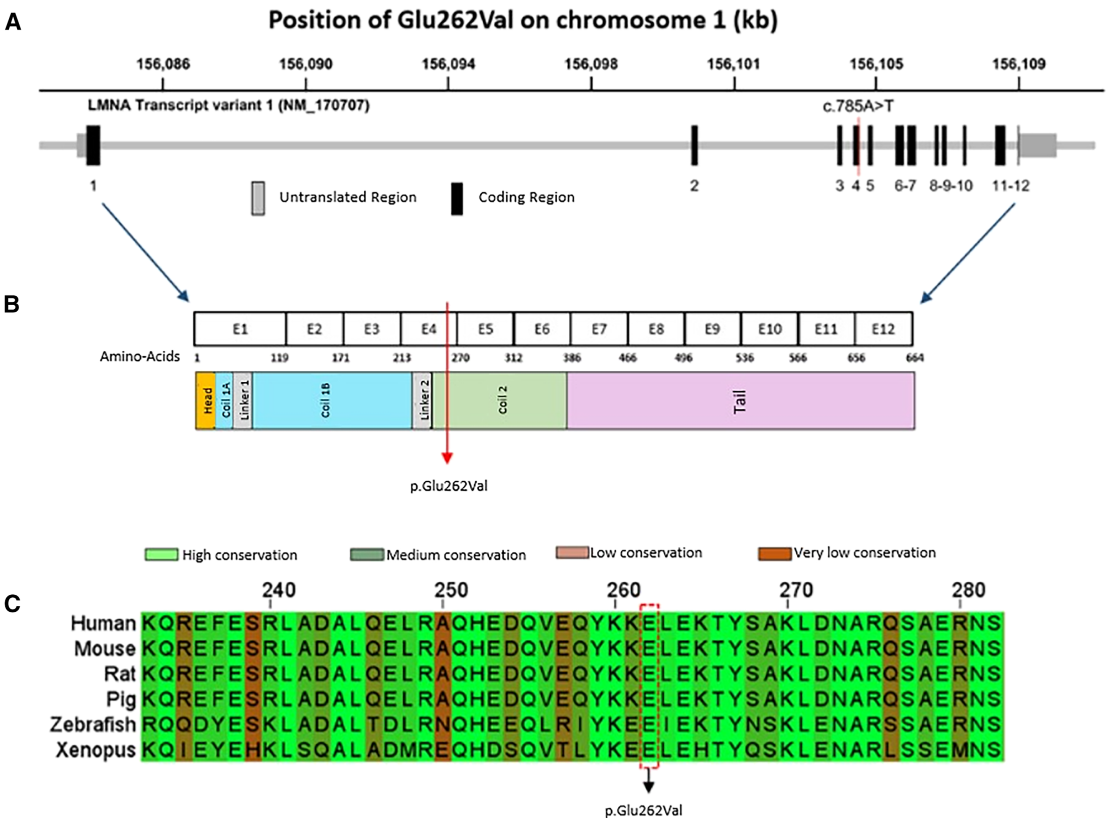

Figure 2. Variant p.Glu262Val in *LMNA*
(A) Exon-intron structure of *LMNA*. Red vertical line indicates the variant p.Glu262Val, c.785A>T.
(B) Corresponding protein domains. Red arrow indicates the p.Glu262Val variant.
(C) Alignment of LMNA amino acid sequences across species. LMNA, Lamin A/C.

mechanisms underpinning the distinct and shared clinical manifestations of the variations is likely to provide more insights about the pathogenesis of valve disease and may help us to guide the development of valve-targeting therapies.

Tissue-specific manifestations of *LMNA* variants is a well-known phenomenon in cardiomyopathies.[21,22] A *LMNA* heterozygous splice-site variant causing cardiac disease showed no molecular or nuclear abnormalities in patient fibroblasts.[23] Reduced *IGFBP5* mRNA levels in these fibroblasts may mask the phenotype in unaffected tissues, while this compensatory mechanism might not occur in cardiac tissues, making them more vulnerable to LMNA haploinsufficiency. Another study evaluated the pro-osteogenic effects of *LMNA* variants in four types of primary human cells of mesenchymal/cardiovascular origin, including human aortic valve interstitial cells.[22] Variant-dependent effects on the expression of osteogenic markers in response to lipopolysaccharide or an osteogenic differentiation medium were observed, but more interestingly the effect of each *LMNA* variant was strongly dependent on the cell type. This raises the possibility that the *LMNA* p.Glu262Val variant may act specifically in heart valve cells, potentially explaining the observed clinical phenotype in affected relatives.

Taken together, the ACMG criteria[15] that support the *LMNA* p.Glu262Val as a likely pathogenic variant and the cause of early onset multivalvular disease in this family are: the candidate missense variant is absent from controls (PM2), novel missense change at an amino acid residue where a different missense change determined to be pathogenic has been seen before (PM5), multiple lines of computational evidence support a deleterious effect on the gene product (PP3), and a low rate of benign missense variant in *LMNA* (PP2). If the nuclear morphology defects observed in our experiments are confirmed by other studies, *in vitro* functional studies supportive of a damaging effect on the protein (PS3) could be added, and the variant classified as pathogenic.

We cannot totally exclude the existence of other disease-causing or disease-modifying variants in this family. We have identified additional candidate variants shared between the proband and the affected father (Table 1). We have also identified a missense *DCHS1* variant (p.S415R). Different variants in this gene have been shown to cause mitral valve prolapse[6] and we previously observed an enrichment of S415R in sporadic cases of mitral valve prolapse.[8] However, its minor allele frequency (1% in individuals of European ancestry) is relatively high making a pure Mendelian disease-causing

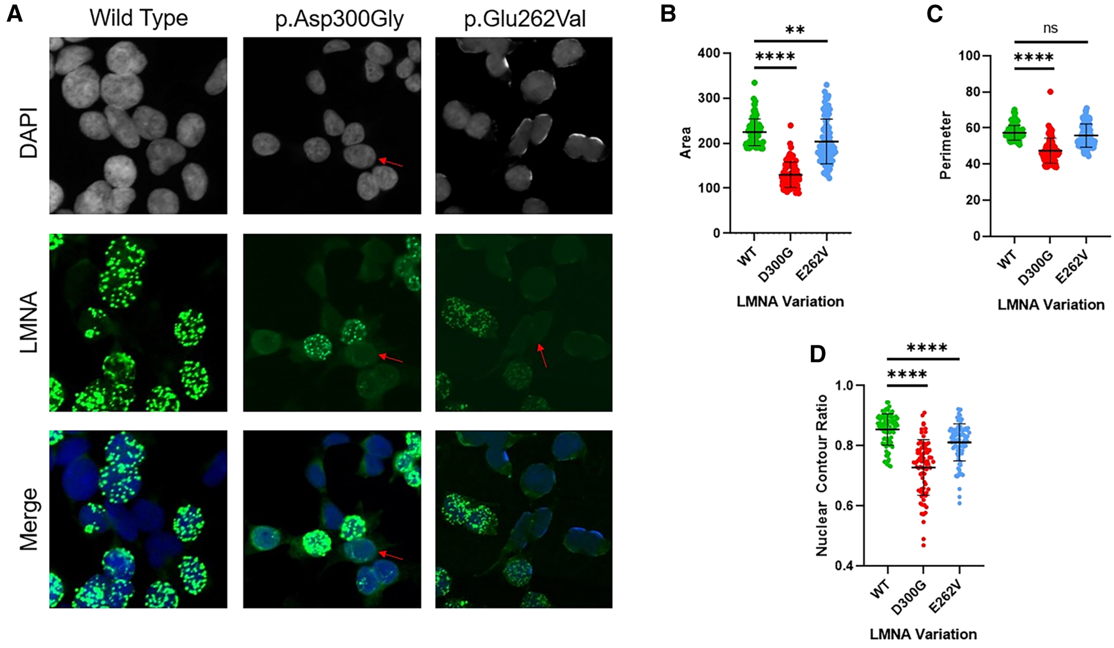

Figure 3. Nuclear abnormalities in p.Glu262Val transfected cells
(A) Fluorescence of nuclei shape from a negative control (wild-type), a positive control (p.D300G) and our variant of interest (p. E262V). Arrows indicate abnormal lamin A/C localization.
(B–D) ImageJ analysis of nuclear morphology for area (B), perimeter (C), and nuclear contour ratio (D). Unpaired t test was performed using GraphPad Prism version 8.0.1 (ns, not significant; $*p < 0.05$, $**p < 0.01$, $***p < 0.001$, $****p < 0.0001$). The results are shown as mean $\pm$ standard deviation.

effect unlikely. Unfortunately, no additional family members are available to delineate the cosegregation of disease with *LMNA* p.Glu262Val, *DCHS1* p.Ser415Arg, and other candidate variants. Thus, a two-hit or multi-hit etiology cannot be excluded in this family.

Some limitations should be mentioned. We reported only one family with few living relatives, restricting the segregation analysis to two affected individuals. The follow-up of the relatives is limited and the curation of variants identified by WES is based on the phenotype observed at the time of the study. We assessed the variant's functional impact via nuclear structure analysis. Since lamin A and GFP-tagged protein overexpression are known to induce aggregates, we analyzed nuclear morphology independently of them. Nonetheless, we cannot fully exclude the possibility that aggregates may have influenced our findings. For this reason, the PS3 argument was not added to our final interpretation.

In conclusion, we identified a *LMNA* missense variant, p.Glu262Val associated with non-syndromic multivalvular disease. It provides insight into the valve diseases pathogenesis and the specificity of p.Glu262Val to cause exclusively cardiac valve manifestations is of interest. Further work is needed to determine the role of *LMNA* in the development of valve diseases and elucidate the targeted impact of p.Glu262Val in valve tissues.

Data and code availability

The datasets supporting the current study have not been deposited in a public repository in accordance with the informed consent and institutional ethics approval, but are available from the corresponding author on request.

Acknowledgments

We are grateful to all members of this family for their proactive collaboration. P.P. holds the Canada Research Chair in Valvular Heart Disease and his research program is supported by a Foundation Scheme Grant from Canadian Institutes of Health Research (Ottawa, Ontario, Canada). P.M. is the recipient of the Joseph C. Edwards Foundation granted to Université Laval. C.S. holds a Junior 1 Clinical Research Scholar award from the Fonds de Recherche du Québec - Santé (FRQS). Y.B. holds a Canada Research Chair in Genomics of Heart and Lung Diseases. This work was supported by the Heart and Stroke Foundation of Canada, the Canadian Institutes of Health Research (PJT–153396, PJT–159641), and the FRQS.

Declaration of interests

The authors declare no competing interests.

Supplemental information

Supplemental information can be found online at https://doi.org/10.1016/j.xhgg.2025.100491.

Received: January 20, 2025
Accepted: August 5, 2025

References

1. Bossé, Y., Mathieu, P., and Thériault, S. (2019). PALMD as a novel target for calcific aortic valve stenosis. Curr. Opin. Cardiol. *34*, 105–111. https://doi.org/10.1097/HCO.0000000000000605.
2. Moncla, L.-H.M., Briend, M., Bossé, Y., and Mathieu, P. (2023). Calcific aortic valve disease: mechanisms, prevention and treatment. Nat. Rev. Cardiol. *20*, 546–559. https://doi.org/10.1038/s41569-023-00845-7.
3. Thériault, S., Li, Z., Abner, E., Luan, J., Manikpurage, H.D., Houessou, U., Zamani, P., Briend, M., Estonian Biobank Research Team, and Boudreau, D.K., et al. (2024). Integrative genomic analyses identify candidate causal genes for calcific aortic valve stenosis involving tissue-specific regulation. Nat. Commun. *15*, 2407. https://doi.org/10.1038/s41467-024-46639-4.
4. Garg, V., Muth, A.N., Ransom, J.F., Schluterman, M.K., Barnes, R., King, I.N., Grossfeld, P.D., and Srivastava, D. (2005). Mutations in NOTCH1 cause aortic valve disease. Nature *437*, 270–274. https://doi.org/10.1038/nature03940.
5. Kyndt, F., Gueffet, J.-P., Probst, V., Jaafar, P., Legendre, A., Le Bouffant, F., Toquet, C., Roy, E., McGregor, L., Lynch, S.A., et al. (2007). Mutations in the gene encoding filamin A as a cause for familial cardiac valvular dystrophy. Circulation *115*, 40–49. https://doi.org/10.1161/CIRCULATIONAHA.106.622621.
6. Durst, R., Sauls, K., Peal, D.S., deVlaming, A., Toomer, K., Leyne, M., Salani, M., Talkowski, M.E., Brand, H., Perrocheau, M., et al. (2015). Mutations in DCHS1 cause mitral valve prolapse. Nature *525*, 109–113. https://doi.org/10.1038/nature14670.
7. Ducharme, V., Guauque-Olarte, S., Gaudreault, N., Pibarot, P., Mathieu, P., and Bossé, Y. (2013). NOTCH1 genetic variants in patients with tricuspid calcific aortic valve stenosis. J. Heart Valve Dis. *22*, 142–149.
8. Clemenceau, A., Bérubé, J.-C., Bélanger, P., Gaudreault, N., Lamontagne, M., Toubal, O., Clavel, M.-A., Capoulade, R., Mathieu, P., Pibarot, P., and Bosse, Y. (2018). Deleterious variants in DCHS1 are prevalent in sporadic cases of mitral valve prolapse. Mol. Genet. Genomic Med. *6*, 114–120. https://doi.org/10.1002/mgg3.347.
9. Lek, M., Karczewski, K.J., Minikel, E.V., Samocha, K.E., Banks, E., Fennell, T., O'Donnell-Luria, A.H., Ware, J.S., Hill, A.J., Cummings, B.B., et al. (2016). Analysis of protein-coding genetic variation in 60,706 humans. Nature *536*, 285–291. https://doi.org/10.1038/nature19057.
10. Adzhubei, I.A., Schmidt, S., Peshkin, L., Ramensky, V.E., Gerasimova, A., Bork, P., Kondrashov, A.S., and Sunyaev, S.R. (2010). A method and server for predicting damaging missense mutations. Nat. Methods *7*, 248–249. https://doi.org/10.1038/nmeth0410-248.
11. Tordai, H., Torres, O., Csepi, M., Padányi, R., Lukács, G.L., and Hegedűs, T. (2024). Analysis of AlphaMissense data in different protein groups and structural context. Sci. Data *11*, 495. https://doi.org/10.1038/s41597-024-03327-8.
12. Kircher, M., Witten, D.M., Jain, P., O'Roak, B.J., Cooper, G. M., and Shendure, J. (2014). A general framework for estimating the relative pathogenicity of human genetic variants. Nat. Genet. *46*, 310–315. https://doi.org/10.1038/ng.2892.
13. Yeo, G., and Burge, C.B. (2004). Maximum entropy modeling of short sequence motifs with applications to RNA splicing signals. J. Comput. Biol. *11*, 377–394. https://doi.org/10.1089/1066527041410418.
14. Jaganathan, K., Kyriazopoulou Panagiotopoulou, S., McRae, J. F., Darbandi, S.F., Knowles, D., Li, Y.I., Kosmicki, J.A., Arbelaez, J., Cui, W., Schwartz, G.B., et al. (2019). Predicting Splicing from Primary Sequence with Deep Learning. Cell *176*, 535–548.e24. https://doi.org/10.1016/j.cell.2018.12.015.
15. Richards, S., Aziz, N., Bale, S., Bick, D., Das, S., Gastier-Foster, J., Grody, W.W., Hegde, M., Lyon, E., Spector, E., et al. (2015). Standards and guidelines for the interpretation of sequence variants: a joint consensus recommendation of the American College of Medical Genetics and Genomics and the Association for Molecular Pathology. Genet. Med. *17*, 405–424. https://doi.org/10.1038/gim.2015.30.
16. Kane, M.S., Lindsay, M.E., Judge, D.P., Barrowman, J., Ap Rhys, C., Simonson, L., Dietz, H.C., and Michaelis, S. (2013). LMNA-associated cardiocutaneous progeria: an inherited autosomal dominant premature aging syndrome with late onset. Am. J. Med. Genet. *161A*, 1599–1611. https://doi.org/10.1002/ajmg.a.35971.
17. Wu, H.W., Van De Peppel, I.P., Rutten, J.W., Jukema, J.W., Aten, E., Jazet, I.M., Koopmann, T.T., Barge-Schaapveld, D. Q.C.M., and Ajmone Marsan, N. (2024). Atypical Progeria Primarily Manifesting as Premature Cardiac Valvular Disease Segregates with LMNA-Gene Variants. JCDD *11*, 86. https://doi.org/10.3390/jcdd11030086.
18. Wilke, M.V.M.B., Wick, M., Schwab, T.L., Starosta, R.T., Clark, K.J., Connolly, H.M., and Klee, E.W. (2024). Nuclear Abnormalities in LMNA p.(Glu2Lys) Variant Segregating with LMNA-Associated Cardiocutaneous Progeria Syndrome. Genes *15*, 112. https://doi.org/10.3390/genes15010112.
19. Janin, A., Bauer, D., Ratti, F., Millat, G., and Méjat, A. (2017). Nuclear envelopathies: a complex LINC between nuclear envelope and pathology. Orphanet J. Rare Dis. *12*, 147. https://doi.org/10.1186/s13023-017-0698-x.
20. Prokocimer, M., Davidovich, M., Nissim-Rafinia, M., Wiesel-Motiuk, N., Bar, D.Z., Barkan, R., Meshorer, E., and Gruenbaum, Y. (2009). Nuclear lamins: key regulators of nuclear structure and activities. J. Cell Mol. Med. *13*, 1059–1085. https://doi.org/10.1111/j.1582-4934.2008.00676.x.
21. Brayson, D., and Shanahan, C.M. (2017). Current insights into LMNA cardiomyopathies: Existing models and missing LINCs. Nucleus *8*, 17–33. https://doi.org/10.1080/19491034.2016.1260798.
22. Perepelina, K., Klauzen, P., Kostareva, A., and Malashicheva, A. (2019). Tissue-Specific Influence of Lamin A Mutations on Notch Signaling and Osteogenic Phenotype of Primary Human Mesenchymal Cells. Cells *8*, 266. https://doi.org/10.3390/cells8030266.
23. Widyastuti, H.P., Norden-Krichmar, T.M., Grosberg, A., and Zaragoza, M.V. (2020). Gene expression profiling of fibroblasts in a family with LMNA-related cardiomyopathy reveals molecular pathways implicated in disease pathogenesis. BMC Med. Genet. *21*, 152. https://doi.org/10.1186/s12881-020-01088-w.

HGGA, Volume *6*

Supplemental information

Early onset multivalvular disease caused

by a missense variant in lamin A/C

Alexandre Janin, Nathalie Gaudreault, Victoria Saavedra Armero, Zhonglin Li, Ran Xu, Dominique K. Boudreau, Lily Frenette, Julien Ternacle, Danielle Tardif, Sébastien Thériault, Philippe Pibarot, Patrick Mathieu, Christian Steinberg, and Yohan Bossé

Supplemental results

Candidate genes testing

Genetic testing was successful to exclude known mutations causing valve diseases in *NOTCH1* *FLNA* and *DCHS1* (**Figure S1**). However, the proband and his father carried a *DCHS1* missense variant in exon 2 (rs117368891, c.1245C>A, p.Ser415Arg), resulting in a substitution of serine with arginine at position 415. Inherited from the father, this S415R variant was absent in unaffected family members, including the mother, paternal aunt, and sister. The proband's heterozygous genotype aligns with dominant inheritance, though segregation data is limited. The strongest evidence of pathogenicity for this variant comes from our previous study[6]. We have recently sequenced the coding regions of *DCHS1* in 100 patients with mitral valve prolapse and three of them were carriers of S415R (3%). This represents an enrichment of 5 to 10-fold compared to reference populations (0.3 to 0.7%). However, the allele frequency is still relatively high (1.08% in non-Finnish Europeans of gnomAD v2.1.1). Some computational algorithms like PolyPhen (1) and CADD (18.93) suggest a deleterious effect, while AlphaMissense classifies it as “ambiguous” (pathogenicity score: 0.408). ClinVar reports the variant six times (ID: 445814), mostly as “benign” or “likely benign”. Overall, the evidence is insufficient to confirm S415R as a disease-causing variant for the patient’s phenotype.

Minigene reporter splicing assay

To evaluate the functional impact on splicing, wild-type (WT) and mutated minigene constructs containing exon 4 of *LMNA* gene and its intronic boundaries were built. After total RNA extraction, RT-PCR amplification of WT and mutated construct resulted in 418 bp products corresponding to normal transcripts, which were further confirmed by Sanger sequencing

(**Figure S4**). Given that the lengths of the transcripts were the same between WT and mutated conditions, the minigene assay confirmed that this exonic variation was not associated with any splicing defects.

A) *NOTCH1*

NOTCH1 R1107X rs41309764	**Proband (son)**	**Homozygote C/C**	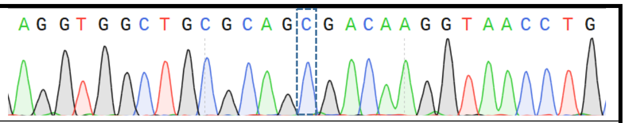
	**Father**	**Homozygote C/C**	Determined by Exome Sequencing
	**Mother**	**Homozygote C/C**	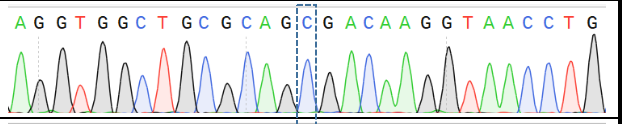
	**Sister**	**Homozygote C/C**	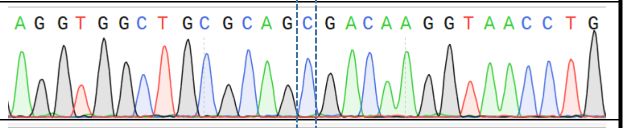
	**Paternal aunt**	**Homozygote C/C**	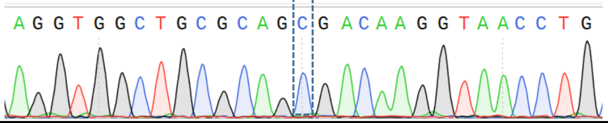

NOTCH1 H1504del rs41309766	**Proband (son)**	**Homozygote G/G**	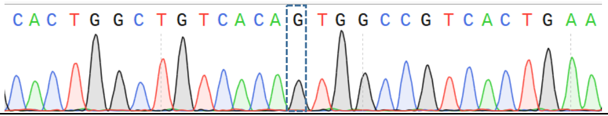
	**Father**	**Homozygote G/G**	Determined by Exome Sequencing
	**Mother**	**Homozygote G/G**	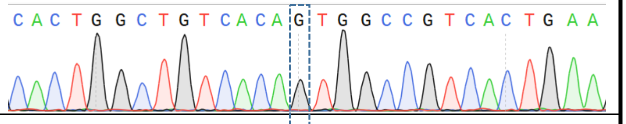
	**Sister**	**Homozygote G/G**	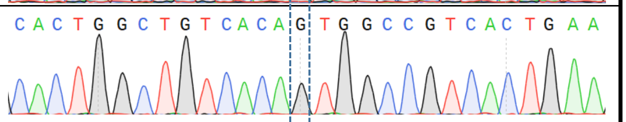
	**Paternal aunt**	**Homozygote G/G**	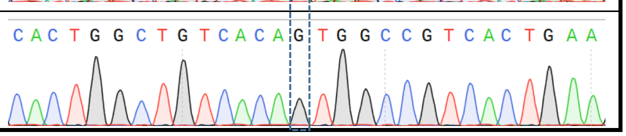

B) *FLNA*

***FLNA* G288R rs267606816**	**Proband (son)**	**Homozygote G/G**	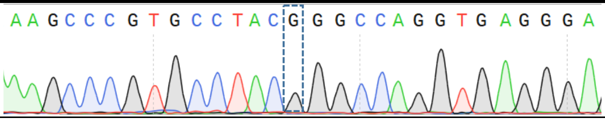
	**Father**	**Homozygote G/G**	Determined by Exome Sequencing
	**Mother**	**Homozygote G/G**	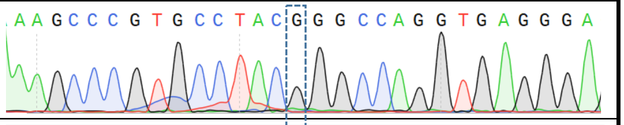
	**Sister**	**Homozygote G/G**	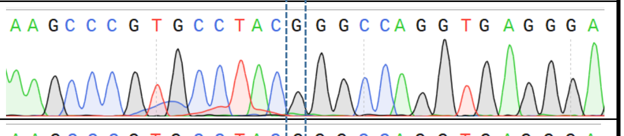
	**Paternal aunt**	**Homozygote G/G**	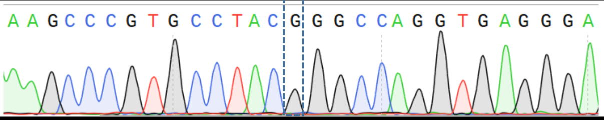

***FLNA* P637Q rs267606815**	**Proband (son)**	**Homozygote G/G**	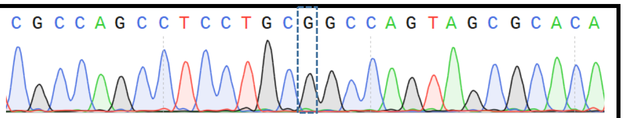
	**Father**	**Homozygote G/G**	Determined by Exome Sequencing
	**Mother**	**Homozygote G/G**	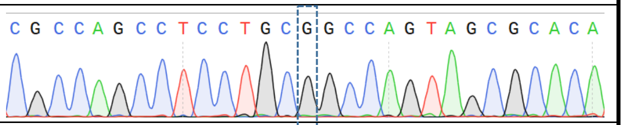
	**Sister**	**Homozygote G/G**	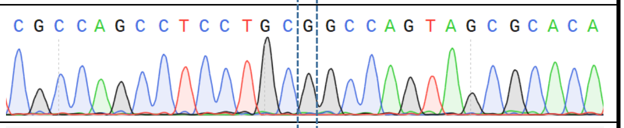
	**Paternal aunt**	**Homozygote G/G**	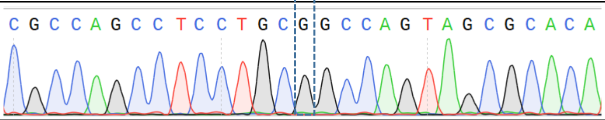

FLNA V711D rs267606817	**Proband (son)**	**Homozygote A/A**	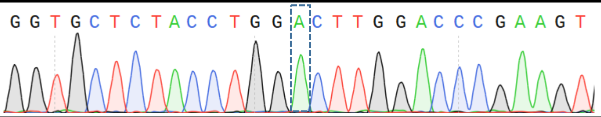
	**Father**	**Homozygote A/A**	Determined by Exome Sequencing
	**Mother**	**Homozygote A/A**	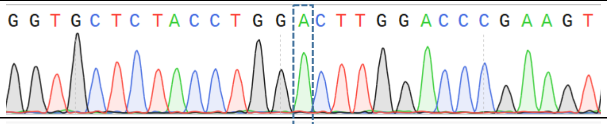
	**Sister**	**Homozygote A/A**	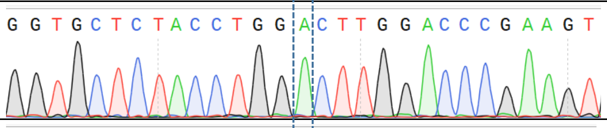
	**Paternal aunt**	**Homozygote A/A**	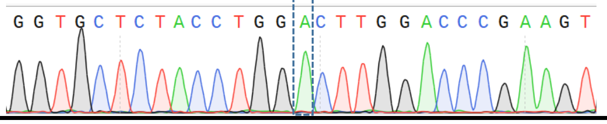

FLNA 1944 bp deletion no rs #	**Father**	**Homozygote 2933 bp**	Determined by Exome Sequencing
	**Proband (son)**	**Homozygote 2933 bp**	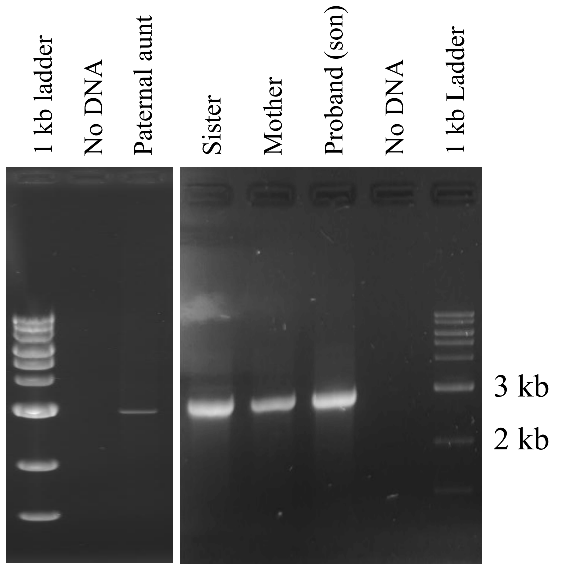
	**Mother**	**Homozygote 2933 bp**	
	**Sister**	**Homozygote 2933 bp**	
	**Paternal aunt**	**Homozygote 2933 bp**	

C) *DCHS1*

***DCHS1* S415R rs117368891**	**Proband (son)**	**Heterozygote G/T**	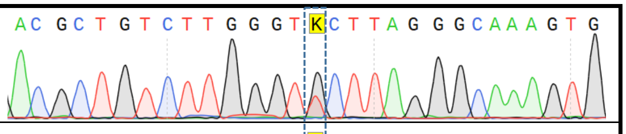
	**Father**	**Heterozygote G/T**	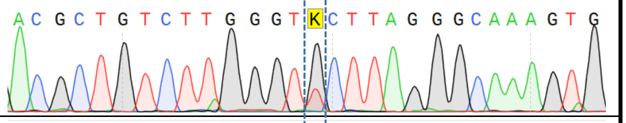
	**Mother**	**Homozygote G/G**	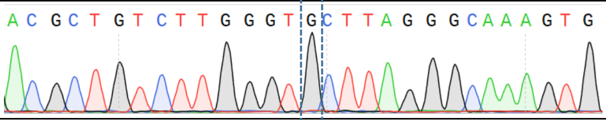
	**Sister**	**Homozygote G/G**	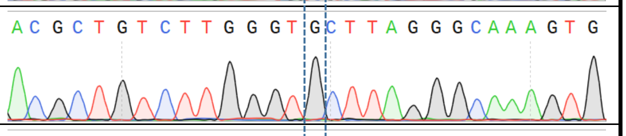
	**Paternal aunt**	**Homozygote G/G**	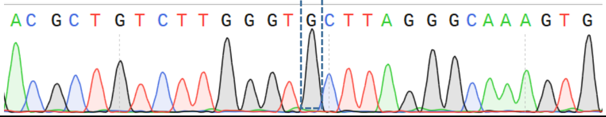

***DCHS1* R2330C rs768737101**	**Proband (son)**	**Homozygote G/G**	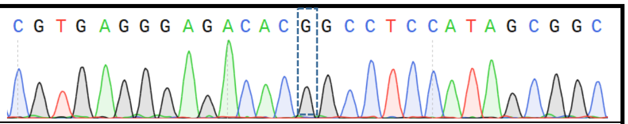
	**Father**	**Homozygote G/G**	Determined by Exome Sequencing
	**Mother**	**Homozygote G/G**	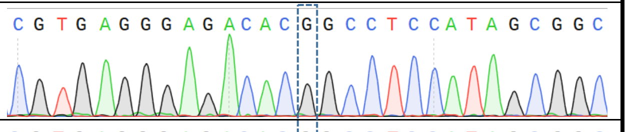
	**Sister**	**Homozygote G/G**	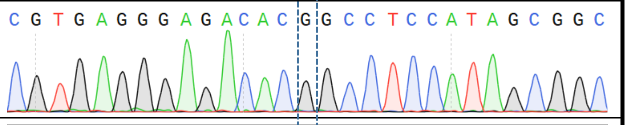
	**Paternal aunt**	**Homozygote G/G**	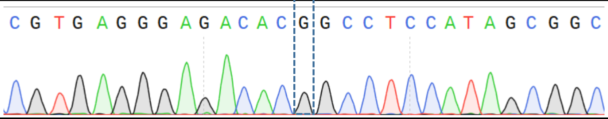

DCHS1 R2462Q rs117140835	**Proband (son)**	**Homozygote G/G**	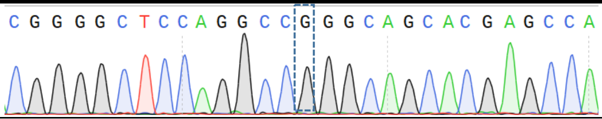
	**Father**	**Homozygote G/G**	Determined by Exome Sequencing
	**Mother**	**Homozygote G/G**	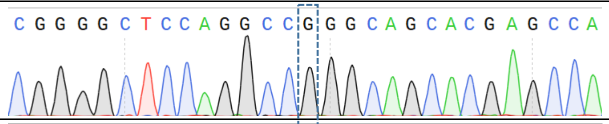
	**Sister**	**Homozygote G/G**	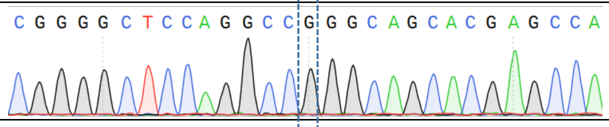
	**Paternal aunt**	**Homozygote G/G**	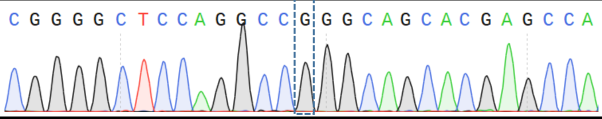

DCHS1 A2464P no rs #	**Proband (son)**	**Homozygote G/G**	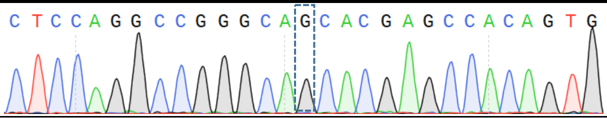
	**Father**	**Homozygote G/G**	Determined by Exome Sequencing
	**Mother**	**Homozygote G/G**	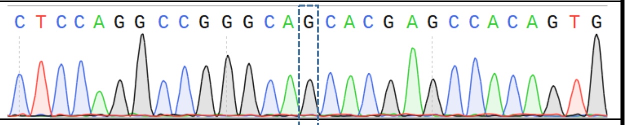
	**Sister**	**Homozygote G/G**	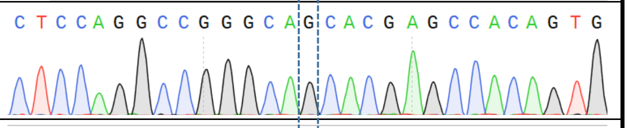
	**Paternal aunt**	**Homozygote G/G**	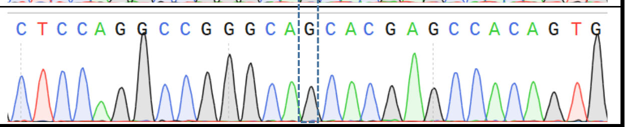

DCHS1 R2513H rs201457110	**Proband (son)**	**Homozygote G/G**	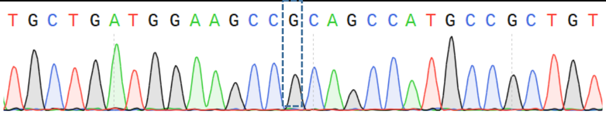
	**Father**	**Homozygote G/G**	Determined by Exome Sequencing
	**Mother**	**Homozygote G/G**	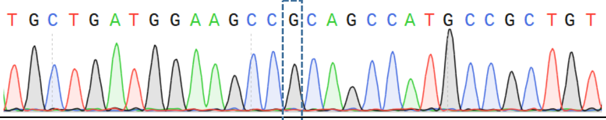
	**Sister**	**Homozygote G/G**	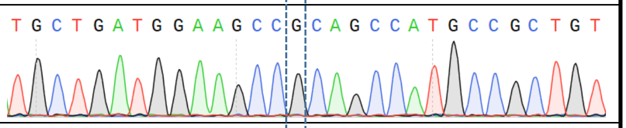
	**Paternal aunt**	**Homozygote G/G**	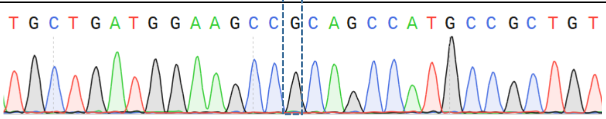

DCHS1 R2770Q rs999967170	**Proband (son)**	**Hom ozygote C/C**	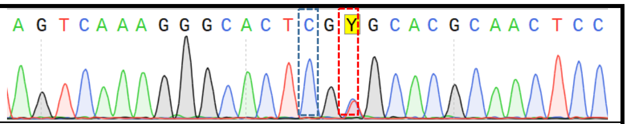
	**Father**	**Homozygote C/C**	Determined by Exome Sequencing
	**Mother**	**Homozygote C/C**	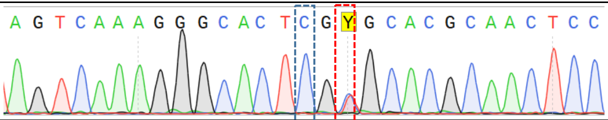
	**Sister**	**Homozygote C/C**	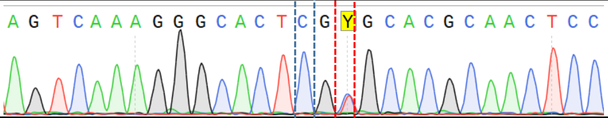
	**Paternal aunt**	**Homozygote C/C**	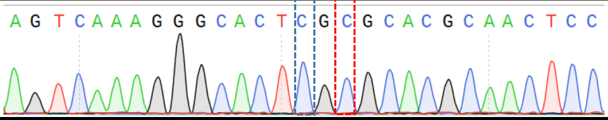
**Note:** Proband, Mother and Sister are heterozygote C/T for synonymous A2769A, rs72911011. Red box			

DCHS1 **R2827P rs35599968**	**Proband (son)**	**Homozygote C/C**	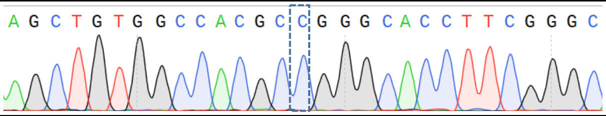
	**Father**	**Homozygote C/C**	Determined by Exome Sequencing
	**Mother**	**Homozygote C/C**	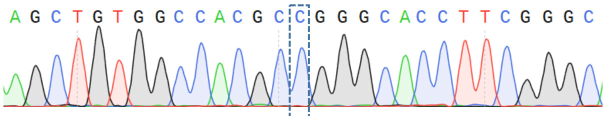
	**Sister**	**Homozygote C/C**	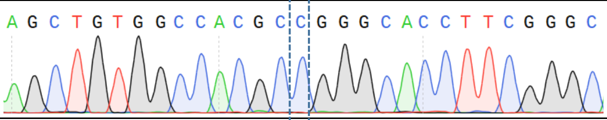
	**Paternal aunt**	**Homozygote C/C**	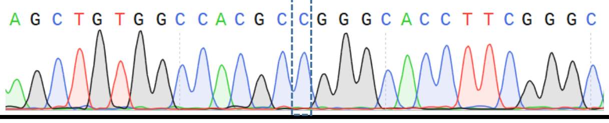

DCHS1 **A2867T rs146233988**	**Proband (son)**	**Homozygote C/C**	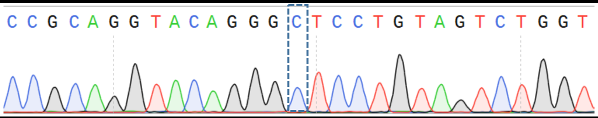
	**Father**	**Homozygote C/C**	Determined by Exome Sequencing
	**Mother**	**Homozygote C/C**	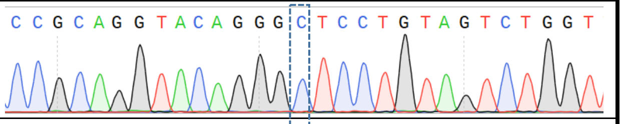
	**Sister**	**Homozygote C/C**	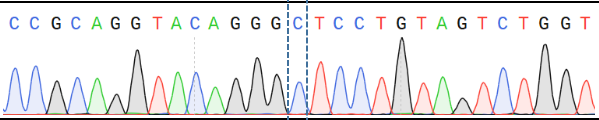
	**Paternal aunt**	**Homozygote C/C**	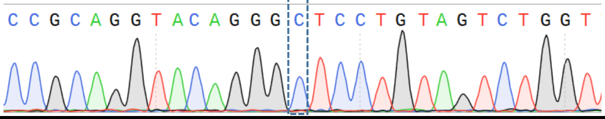

Figure S1. Sanger sequencing results of candidate mutations causing valve diseases in *NOTCH1* (A), *FLNA* (B), and *DCHS1* (C).

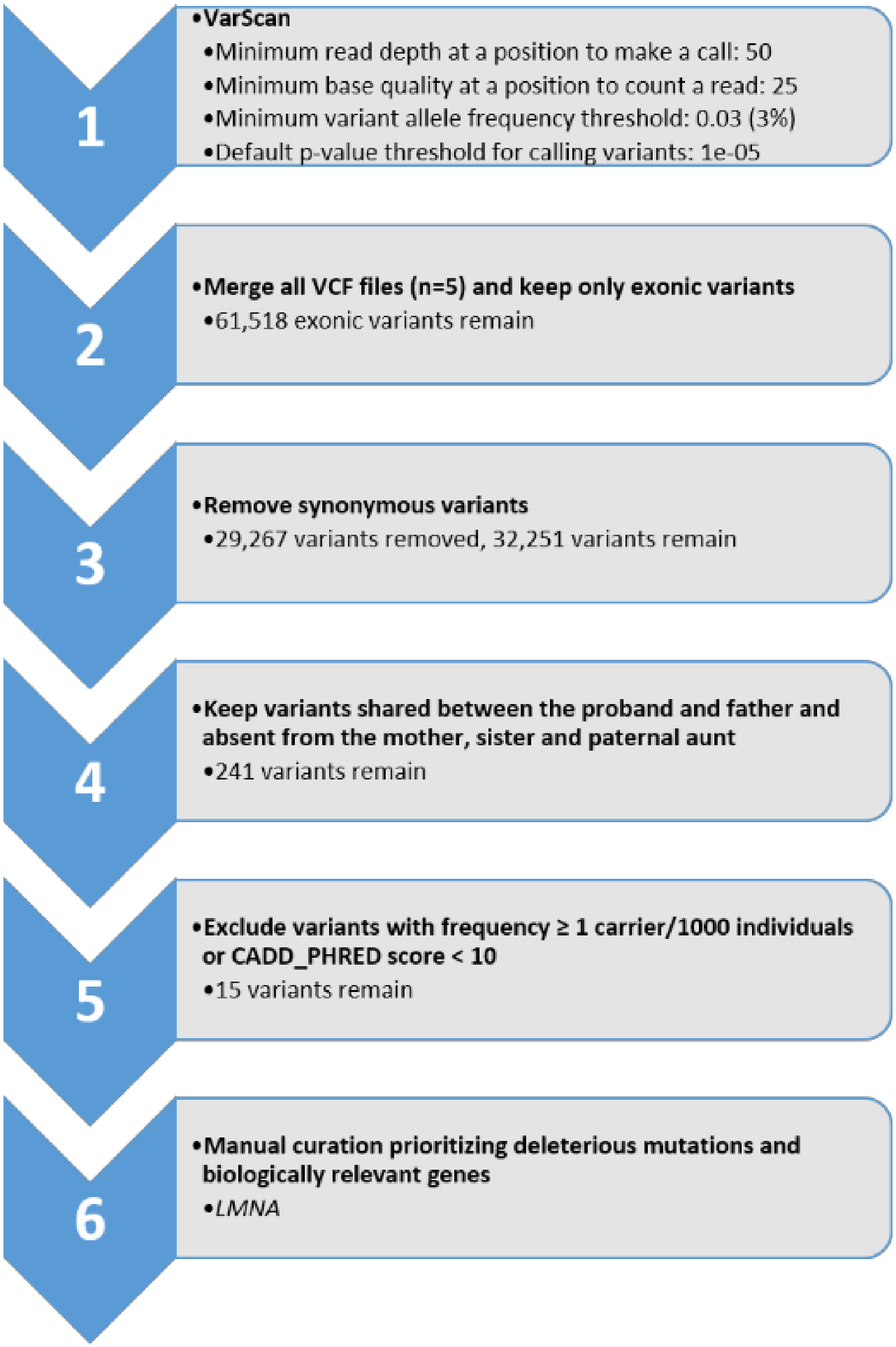

Figure S2. Steps to filter genetic variants identified by whole-exome sequencing.

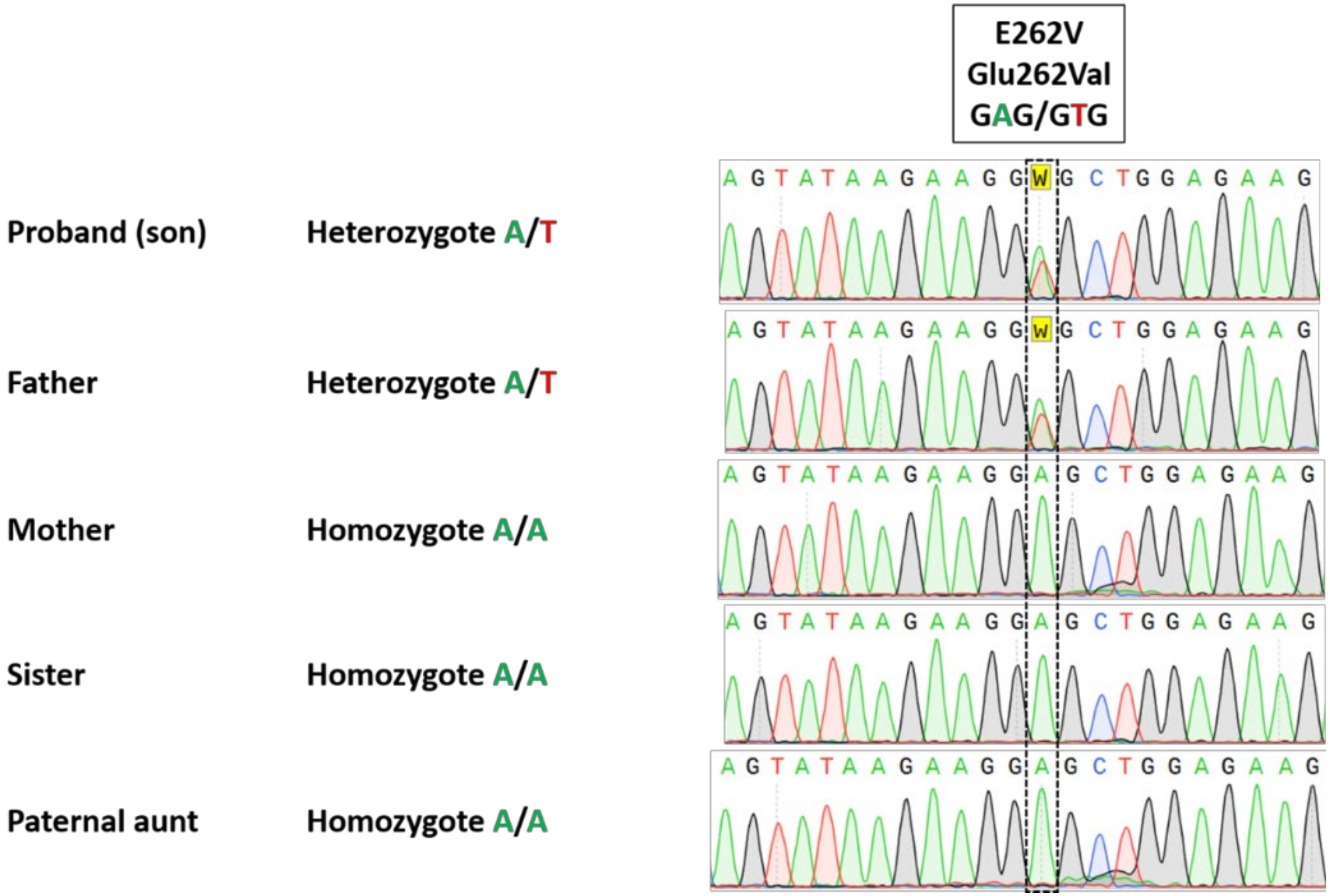

Figure S3. Sanger sequencing confirmation of p.Glu262Val in *LMNA*.

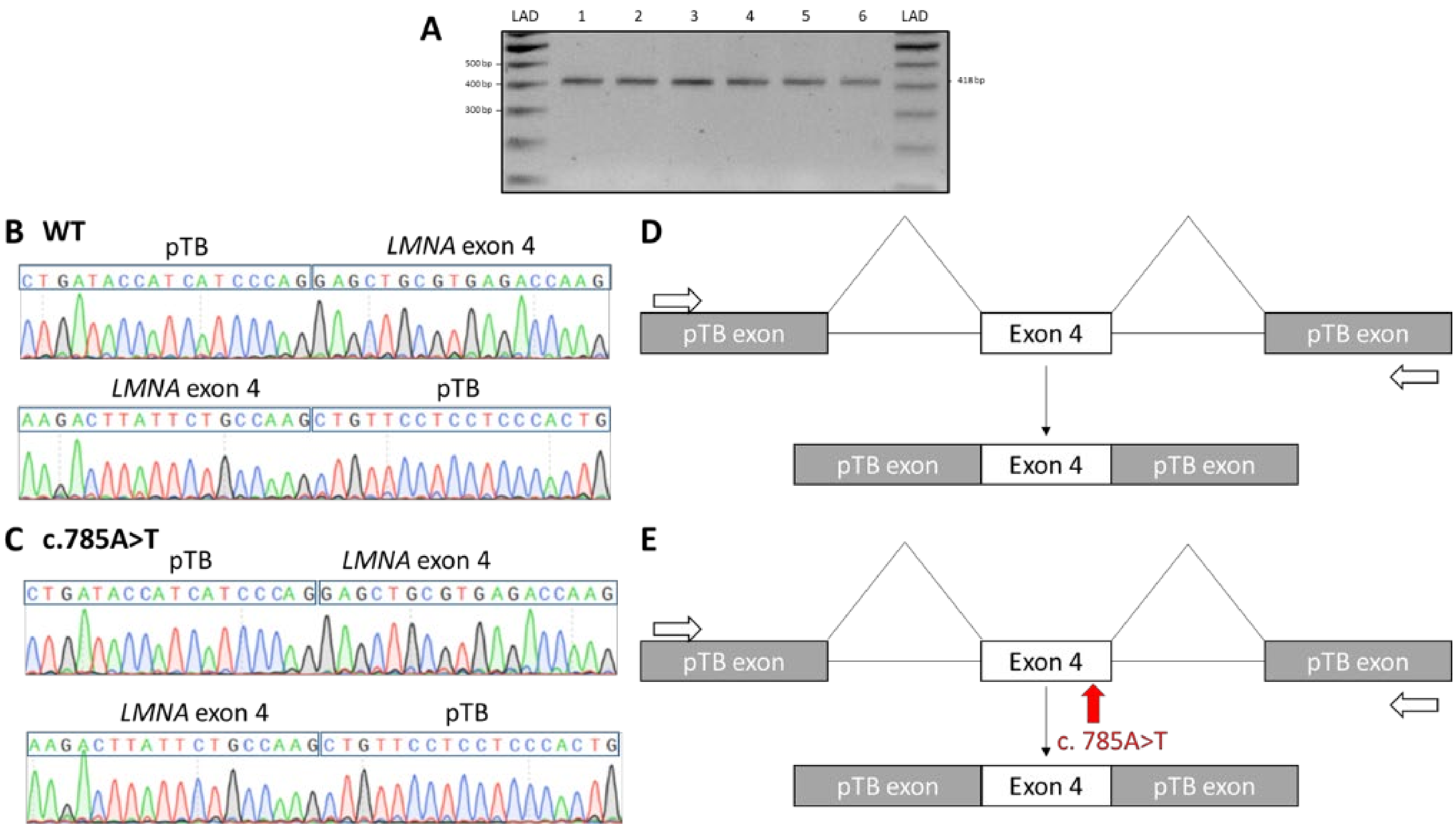

Figure S4. Minigene splicing assay results.

(A) RT-PCR results obtained after transfection of HeLa cells with wild-type (lanes 1 to 3) and mutated (lanes 4 to 6) constructs for *LMNA*: c.785A>T. (B-C) Sanger sequencing results of the product obtained after transfection with wild-type (B) and mutated (C) constructs, using pTB minigene vector. (D-E) Schematic representations of the minigene including *LMNA* exon 4 (white boxes) and intronic regions (black lines) for the wild-type (D) and the mutated (E) constructs.

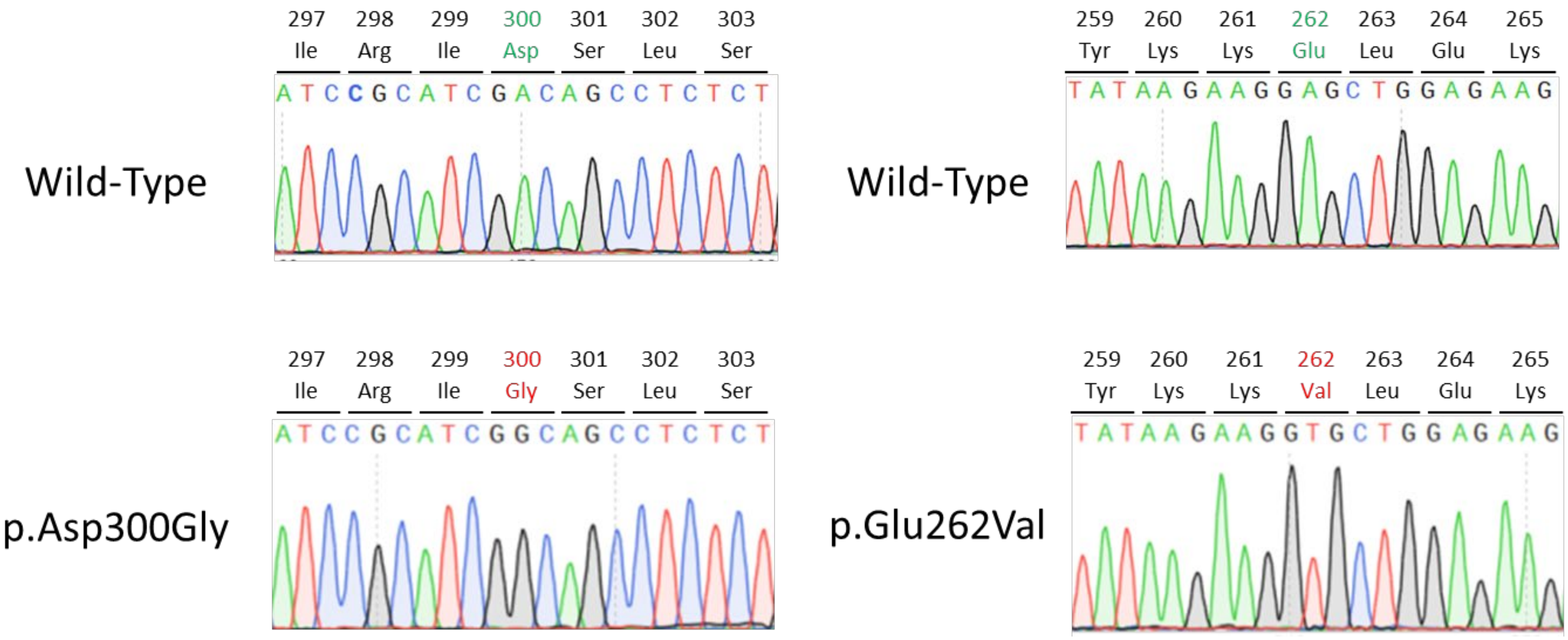

Figure S5. Sanger sequencing results of plasmids.

Nucleotide change	Effect on protein	GnomAD v4		Prediction Algorithms			Domain	Familial Study	Functional study	ACMG criteria for pathogenicity	ACMG	Ref
		n	%	CADD	AlphaMissense	REVEL						
c.4G>A	p.(Glu2Lys)	0	0	24,0	0,85<br>Likely Pathogenic	0,351<br>Uncertain	Head	Yes	Yes	PS3, PM1, PM2, PP1	LP	6
c.11C>G	p.(Pro4Arg)	0	0	24,3	0,585<br>Likely Pathogenic	0,589<br>Damaging	Head	Yes	Yes	PS3, PP5, PM1, PM5, PP3, PM2	P	7,8
c.29C>T	p.(Thr10Ile)	0	0	23,0	0,356<br>Ambiguous	0,539<br>Damaging	Head	Yes	Yes	PS3, PP5, PM1, PM5, PM2	P	8
c.175C>G	p.(Leu59Val)	0	0	26,4	0,978<br>Likely Pathogenic	0,898<br>Damaging	Coil 1A	No	No	PM1, PP3, PM5, PM2	LP	7
c.331G>A	p.(Glu111Lys)	0	0	29,4	0,916<br>Likely Pathogenic	0,779<br>Damaging	Coil 1B	Yes	Yes	PS3, PM1, PM2, PP5	LP	8
c.398G>T	p.(Arg133Leu)	0	0	25,0	0,775<br>Likely Pathogenic	0,811<br>Damaging	Coil 1B	Yes	Yes	PS3, PM5, PP3, PP5, PM1, PM2	P	9,10
c.406G>C	p.(Asp136His)	0	0	28,3	0,943<br>Likely Pathogenic	0,838<br>Damaging	Coil 1B	Yes	Yes	PM5, PM1, PP3, PM2	LP	8
c.412G>C	p.(Glu138Gln)	0	0	29,2	0,708<br>Likely Pathogenic	0,73<br>Damaging	Coil 1B	Yes	No	PM2, PP3, PP1	VUS	11
c.412G>A	p.(Glu138Lys)	0	0	32,0	0,970<br>Likely Pathogenic	0,915<br>Damaging	Coil 1B	Yes	No	PP3, PP5, PM1, PM2	P	12
c.419T>G	p.(Leu140Arg)	0	0	23,5	0,537<br>Ambiguous	0,702<br>Damaging	Coil 1B	No	Yes	PS3, PM1, PM5, PM2, PP5	P	13
c.434A>G	p.(Glu145Gly)	0	0	33,0	0,851<br>Likely Pathogenic	0,764<br>Damaging	Coil 1B	Yes	No	PM2, PP3, PP1	VUS	11
c.475G>A	p.(Glu159Lys)	0	0	24,7	0,614<br>Likely Pathogenic	0,773<br>Damaging	Coil 1B	Yes	Yes	PS3, PP3, PM1, PM2	LP	8
**c.785A>T**	**p.(Glu262Val)**	**0**	**0**	**33,0**	**0,945<br>Likely Pathogenic**	**0,877<br>Damaging**	**Coil 2**	**Yes**	**Yes**	**(PP1), (PM1) PS3, PM2, PM5, PP3**	**LP**	**This study**
c.898G>C	p.(Asp300His)	0	0	28,3	0,972<br>Likely Pathogenic	0,830<br>Damaging	Coil 2	Yes	No	PM5, PM1, PP3, PM2	LP	14
c.898G>A	p.(Asp300Asn)	0	0	28,8	0,821<br>Likely Pathogenic	0,606<br>Damaging	Coil 2	Yes	No	PP5, PM1, PM5, PP3, PM2	LP	15
c.899A>G	p.(Asp300Gly)	0	0	24,4	0,923<br>Likely Pathogenic	0,833<br>Damaging	Coil 2	Yes	Yes	PS3, PM1, PM5, PP3, PM2, PP5	P	16

Table S1. List of previously reported *LMNA* variants in patients with valvular phenotype.

Nucleotide change	Effect on protein	Clinical Features	Valvular Features	Premature aging	Metabolic abnormalities	Skin Changes	Neuromuscular Phenotype	Ref
c.4G>A	p.(Glu2Lys)	LCPS	severe calcific aortic stenosis, calcified mitral valve	Yes	No	No	No	6
c.11C>G	p.(Pro4Arg)	APS	Mitral and Aortic Valve Replacement	NR	Partial or Generalized Lipodystrophy	Yes	NR	7,8
c.29C>T	p.(Thr10Ile)	APS	Aortic Stenosis	NR	Diabetes and Generalized Lipodystrophy	Yes	NR	8
c.29C>T	p.(Thr10Ile)	APS	Mitral, Aortic and Tricuspid Regurgitation	NR	Diabetes and Generalized Lipodystrophy	Yes	NR	8
c.175C>G	p.(Leu59Val)	APS	Tricuspid Regurgitation	Yes	Partial Lipodystrophy	Yes	No	17
c.331G>A	p.(Glu111Lys)	APS	Tricuspid Regurgitation	NR	No	Yes	NR	8
c.398G>T	p.(Arg133Leu)	Atypical WS	Thickened valves, aortic regurgitation	Yes	Generalized Lipoatrophy, massive liver steatosis, hypertriglyceridemia, diabetes	Yes	No	9,10
c.406G>C	p.(Asp136His)	APS	Mitral and Aortic Valve Replacement	NR	Diabetes and Partial Lipodystrophy	Yes	NR	8
c.412G>C	p.(Glu138Gln)	Valvular features only	Premature aortic and mitral valve calcification, Severe Aortic Stenosis	No	No	No	No	11
c.412G>A	p.(Glu138Lys)	APS	Mitral, tricuspid and aortic incompetence	Yes	NR	Yes	Yes	12
c.419T>G	p.(Leu140Arg)	Atypical WS	Aortic Stenosis	Yes	No	Yes	NR	13
c.434A>G	p.(Glu145Gly)	Valvular features only	Severe Aortic and mitral valve stenosis and calcifications	No	No	No	No	11
c.475G>A	p.(Glu159Lys)	APS	Mitral and Aortic Valve Replacement	NR	No	Yes	NR	8
**c.785A>T**	**p.(Glu262Val)**	**Valvular features only**	**Severe aortic valve and mitral annulus calcification**	**No**	**No**	**No**	**No**	**This study**
c.898G>C	p.(Asp300His)	Atypical WS	Mitral Calcifications	Yes	Lipoatrophy	Yes	No	14
c.898G>A	p.(Asp300Asn)	Atypical WS	Aortic valve calcifications	Yes	NR	Yes	No	15
c.899A>G	p.(Asp300Gly)	LCPS	Mitral calcification and regurgitation, Aortic valve stenosis	Yes	NR	Yes	NR	16

Table S2. Detailed phenotypes of reported patients with *LMNA* variants associated with valvular abnormalities.

APS	Atypical Progeroid Syndrome
LCPS	*LMNA*-associated cardiocutaneous progeria syndrome
NR	Not Reported
WS	Werner Syndrome

Gene	Variation tested	rs #	Forward	Reverse	PCR *	Amplicon Size (bp)
**PCR and Sanger Sequencing Primers**						
NOTCH1	R1107X	rs41309764	5’-GTCCACCAGGTCCTCACAGT-3’	**5’-TGTGCACTGGTGTGACTCCT-3’**	**A**	357
NOTCH1	H1504del	rs41309766	5’-AAAGGGTTTTGCTGCTGGGG-3’	**5’-CCCCTGAGCAGAGCCTTAGA-3’**	**A**	822
FLNA	G288R	rs267606816	**5’-AACGTGGACGAGCACTCTGTC-3’**	5’-CTCTTGGGTGGCTTGATCACCTAG-3’	**A**	247
FLNA	P637Q<br>V711D	rs267606815<br>rs267606817	5’-TCGCAGGCTAAGATCGAATGTGAC-3’	**5’-GTAGGAGCAGCTGTAAGTGCCATTG-3’**	**B**	603
FLNA	1944del	no rs #	5’-ACCTGCTCCTATCTGCCTGACAG-3’	5’-CCTCAGAGAGCACAGTGGGTTC-3’	**E**	2933 or 989
DCHS1	S415R	rs117368891	5’-CAATGTGTCCCTGGAAGGTGGAGA-3’	5’-CCTGCCAAGACCATCTGCCTC-3’	**C**	815
				**5’-GGAGGTGAGCCTGAGTCTGTG-3’**		Sequencing
DCHS1	R2330C	rs768737101	5’-TCTCCACCCTGCAGCTCAAG-3’	5’-ACTCAGGGAATGGCCTATCTGCT-3’	**C**	1058
				**5’-GTAGTTCACAGGACTTGGGACC-3’**		Sequencing
DCHS1	R2462Q<br>A2464P<br>R2513H<br>R2770Q<br>R2827P<br>A2867T	rs117140835<br>no rs #<br>rs201457110<br>rs999967170<br>rs35599968<br>rs146233988	5’-**AGCAGATAGGCCATTCCCTGAGT**-3’	**5’-CAGCTCCTGGCCCACCATAG-3’**	**D**	1883
				**5’-CATCCTCATCTGTGGCCTGC-3’**		Sequencing
LMNA	E262V	no rs #	5’-CAGCATGAGGACCAGGTGGAG-3’	**5’-CCAGAAGGCATAGCCCAGC-3’**	**C**	140
**Minigene Assay Primers**						
LMNA	EXON 4		5’-CTAAACAGCCACATATGTGTGCAGAGCTCGCCTTC -3’	5’-CCCCCCTCGACCATATGCCTGAGTTGGGCATCACTG -3’	**F**	523
**Site-directed Mutagenesis Primers**						
LMNA	E262V		5’-TATAAGAAGGTGCTGGAGAAGACTTATTCTG -3’	5’- CTGCTCCACCTGGTCCTC -3’		
LMNA	D300G		5’-ATCCGCATCGGCAGCCTCTCTG-3’	5’-GCGCGACTGCTGCAGCTC-3’		

Sequencing primers are shown in bold.
*Refer to Supplementary Table S4 for PCR cycling conditions.

Table S3. Primers to sequence mutations causing valve diseases in the *NOTCH1*, *FLNA* and *DCHS1* genes and to perform site-directed mutagenesis.

PCR condition	Initial Denaturation	Denaturation	Annealing	Elongation
**A**	95°C, 15 min	94°C, 15 sec	57.0°C, 30 sec	72°C, 1 min
**B**	95°C, 15 min	94°C, 15 sec	60.0°C, 30 sec	72°C, 1 min
**C**	95°C, 15 min	94°C, 15 sec	59.0°C, 30 sec	72°C, 1 min
**D**	95°C, 15 min	94°C, 15 sec	59.0°C, 30 sec	72°C, 2 min
**E**	95°C, 15 min	94°C, 15 sec	59.0°C, 30 sec	72°C, 3 min
**F**	98°C, 30 sec	98°C, 10 sec	55.0°C, 30 sec	72°C, 30 sec

Table S4. PCR cycling conditions.

Phenotype	HPO number
Aortic valve calcification	HP:0004380
Mitral annulus calcification	HP:0005136
Aortic valve stenosis	HP:0001650
Mitral valve regurgitation	HP:0001653

Table S5. Human Phenotype Ontology (HPO) annotations of the reported phenotype findings.

Supplemental materials

Samples and DNA extraction

Blood samples were collected in EDTA tubes. After centrifugation, buffy coats were collected and stored at -80°C. DNA from four individuals (proband, mother, sister and paternal aunt) was extracted using the QIAamp DNA Blood Mini kit (Qiagen). DNA from the skin melanoma biopsy (father) was extracted using QIAamp DNA FFPE Tissue kit (Qiagen). The DNA was further purified using Micro Bio-Spin Chromatography-6 Column (Bio-Rad) in order to remove melanin from skin which inhibits polymerase activity. The DNA quality and concentration were assessed by the UV absorbance ratio 260/280 nm and UV absorbance 260 nm, respectively.

Sanger sequencing of candidate genes

Three genes known to cause non-syndromic valve diseases were evaluated, namely *NOTCH1*, *FLNA*, and *DCHS1*. Primers were designed to cover mutations R1107X and H1504del for *NOTCH1*[1]; G288R, P637Q, V711D and 1944bp_del for *FLNA*[2]; and S415R, R2330C, R2462Q, A2464P, R2513H, R2770Q, R2827P and A2867T for *DCHS1*[3,4]. Primer sequences are provided in **Table S3**. PCR was performed in a final volume of 25 µL containing 100 ng of genomic DNA, 1 U of HotStarTaq DNA polymerase (Qiagen), PCR buffer 1X, Q-Solution 1X, 160 µM of each dNTP and 0.2 µM of each primer. The PCR reaction was carried out on the GeneAmp® PCR system 9700 (Applied Biosystems). PCR cycling conditions are provided in **Table S4**. The sequencing reaction was then performed using standard procedures and the product was run on the ABI 3730xl DNA Analyzer (Applied Biosystems). Sequencing files were assembled and analyzed using the EMBL-EBI Clustal Omega Multiple Alignment Tool

(http://www.ebi.ac.uk/Tools/msa/clustalo). For the deletion of 1944 bp in *FLNA*, the DNA fragment was amplified and separated by electrophoresis using 1% agarose gel (**Figure S1**).

Minigene Splicing Reporter Assay

Minigene Splicing reporter assay was performed as previously described [5]. Briefly, a genomic region spanning exon 4 of *LMNA* (227bp in intron 3 and 154bp in intron 4) was amplified from the proband DNA using the Q5® High-Fidelity DNA Polymerase (New England Biolabs) according to the manufacturer`s instructions. The primers used are described in **Table S3**. Amplicons were inserted in the *NdeI* restriction site of the previously described pTB minigene vector using the In-fusion Snap Assembly cloning kit (Takara Bio). DNA sequences of wild-type and mutant plasmids were verified by Sanger sequencing. HeLa cells were plated at a concentration of 5×10^4 cells/well in a 12-well cluster plate in 1ml of growth medium and transiently transfected with 1 μg of plasmid using JetOPTIMUS® (PolyPlus) one day after seeding following the manufacturer's recommendations. Forty-eight hours after transfection, total RNA was extracted from the cells using RNeasy Plus Micro Kit (Qiagen). RT-PCR (reverse transcription polymerase chain reaction) was performed with Quantitect Reverse Transcription Kit (Qiagen) using 250 ng of total RNA and random primers. Complementary DNA (cDNA) amplification was performed using vector-specific primers surrounding the cloning site and HotStar Taq Plus DNA polymerase (Qiagen). The PCR products were resolved on a 2% agarose gel and Sanger sequenced to identify splicing events. All transfection experiments were performed in triplicate.

Construction of vectors

The cDNA constructs harboring the E262V and D300G mutations were verified by Sanger sequencing (**Figure S5**) and primers are listed in **Table S3**.

Supplemental References

1. Garg, V., Muth, A.N., Ransom, J.F., Schluterman, M.K., Barnes, R., King, I.N., Grossfeld, P.D., and Srivastava, D. (2005). Mutations in NOTCH1 cause aortic valve disease. Nature *437*, 270–274. https://doi.org/10.1038/nature03940.

2. Kyndt, F., Gueffet, J.-P., Probst, V., Jaafar, P., Legendre, A., Le Bouffant, F., Toquet, C., Roy, E., McGregor, L., Lynch, S.A., et al. (2007). Mutations in the gene encoding filamin A as a cause for familial cardiac valvular dystrophy. Circulation *115*, 40–49. https://doi.org/10.1161/CIRCULATIONAHA.106.622621.

3. Durst, R., Sauls, K., Peal, D.S., deVlaming, A., Toomer, K., Leyne, M., Salani, M., Talkowski, M.E., Brand, H., Perrocheau, M., et al. (2015). Mutations in DCHS1 cause mitral valve prolapse. Nature *525*, 109–113. https://doi.org/10.1038/nature14670.

4. Clemenceau, A., Bérubé, J.-C., Bélanger, P., Gaudreault, N., Lamontagne, M., Toubal, O., Clavel, M.-A., Capoulade, R., Mathieu, P., Pibarot, P., et al. (2018). Deleterious variants in DCHS1 are prevalent in sporadic cases of mitral valve prolapse. Mol. Genet. Genomic Med. *6*, 114–120. https://doi.org/10.1002/mgg3.347.

5. Janin, A., Chanavat, V., Rollat-Farnier, P.-A., Bardel, C., Nguyen, K., Chevalier, P., Eicher, J.-C., Faivre, L., Piard, J., Albert, E., et al. (2020). Whole MYBPC3 NGS sequencing as a molecular strategy to improve the efficiency of molecular diagnosis of patients with hypertrophic cardiomyopathy. Hum. Mutat. *41*, 465–475. https://doi.org/10.1002/humu.23944.

6. Wilke, M.V.M.B., Wick, M., Schwab, T.L., Starosta, R.T., Clark, K.J., Connolly, H.M., and Klee, E.W. (2024). Nuclear Abnormalities in LMNA p.(Glu2Lys) Variant Segregating with LMNA-Associated Cardiocutaneous Progeria Syndrome. Genes *15*, 112. https://doi.org/10.3390/genes15010112.

7. Guo, H., Luo, N., Hao, F., and Bai, Y. (2014). p.Pro4Arg mutation in LMNA gene: a new atypical progeria phenotype without metabolism abnormalities. Gene *546*, 35–39. https://doi.org/10.1016/j.gene.2014.05.042.

8. Garg, A., Subramanyam, L., Agarwal, A.K., Simha, V., Levine, B., D'Apice, M.R., Novelli, G., and Crow, Y. (2009). Atypical progeroid syndrome due to heterozygous missense LMNA mutations. J. Clin. Endocrinol. Metab. *94*, 4971–4983. https://doi.org/10.1210/jc.2009-0472.

9. Caux, F., Dubosclard, E., Lascols, O., Buendia, B., Chazouillères, O., Cohen, A., Courvalin, J.-C., Laroche, L., Capeau, J., Vigouroux, C., et al. (2003). A new clinical condition linked to a novel mutation in lamins A and C with generalized lipoatrophy, insulin-resistant diabetes, disseminated leukomelanodermic papules, liver steatosis, and cardiomyopathy. J. Clin. Endocrinol. Metab. *88*, 1006–1013. https://doi.org/10.1210/jc.2002-021506.

10. Jacob, K.N., Baptista, F., dos Santos, H.G., Oshima, J., Agarwal, A.K., and Garg, A. (2005). Phenotypic heterogeneity in body fat distribution in patients with atypical Werner's

syndrome due to heterozygous Arg133Leu lamin A/C mutation. J. Clin. Endocrinol. Metab. *90*, 6699–6706. https://doi.org/10.1210/jc.2005-0939.

11. Wu, H.W., Van De Peppel, I.P., Rutten, J.W., Jukema, J.W., Aten, E., Jazet, I.M., Koopmann, T.T., Barge-Schaapveld, D.Q.C.M., and Ajmone Marsan, N. (2024). Atypical Progeria Primarily Manifesting as Premature Cardiac Valvular Disease Segregates with LMNA-Gene Variants. J. Cardiovasc. Dev. Dis. *11*, 86. https://doi.org/10.3390/jcdd11030086.

12. Doubaj, Y., De Sandre-Giovannoli, A., Vera, E., Navarro, C.L., Elalaoui, S.C., Tajir, M., Lévy, N., and Sefiani, A. (2012). An inherited *LMNA* gene mutation in atypical Progeria syndrome. Am. J. Med. Genet. A. *158A*, 2881–2887. https://doi.org/10.1002/ajmg.a.35557.

13. Chen, L., Lee, L., Kudlow, B.A., Dos Santos, H.G., Sletvold, O., Shafeghati, Y., Botha, E.G., Garg, A., Hanson, N.B., Martin, G.M., et al. (2003). LMNA mutations in atypical Werner's syndrome. The Lancet *362*, 440–445. https://doi.org/10.1016/S0140-6736(03)14069-X.

14. Yanhua, X., and Suxian, Z. (2018). Cerebral Haemorrhage in a Young Patient With Atypical Werner Syndrome Due to Mutations in LMNA. Front. Endocrinol. *9*, 433. https://doi.org/10.3389/fendo.2018.00433.

15. Renard, D., Fourcade, G., Milhaud, D., Bessis, D., Esteves-Vieira, V., Boyer, A., Roll, P., Bourgeois, P., Levy, N., and De Sandre-Giovannoli, A. (2009). Novel *LMNA* Mutation in Atypical Werner Syndrome Presenting With Ischemic Disease. Stroke *40*. https://doi.org/10.1161/STROKEAHA.108.531780.

16. Kane, M.S., Lindsay, M.E., Judge, D.P., Barrowman, J., Ap Rhys, C., Simonson, L., Dietz, H.C., and Michaelis, S. (2013). LMNA-associated cardiocutaneous progeria: an inherited autosomal dominant premature aging syndrome with late onset. Am. J. Med. Genet. A. *161A*, 1599–1611. https://doi.org/10.1002/ajmg.a.35971.

17. Guo, X., Ling, C., Liu, Y., Zhang, X., and Zhang, S. (2016). A Case of Novel Lamin A/C Mutation Manifesting as Atypical Progeroid Syndrome and Cardiomyopathy. Can. J. Cardiol. *32*, 1166.e29-31. https://doi.org/10.1016/j.cjca.2015.11.011.
